# Tuning the Size of TiO_2_-Supported Co Nanoparticle Fischer–Tropsch Catalysts Using Mn Additions

**DOI:** 10.1021/acscatal.4c02721

**Published:** 2024-07-01

**Authors:** Matthew Lindley, Pavel Stishenko, James W. M. Crawley, Fred Tinkamanyire, Matthew Smith, James Paterson, Mark Peacock, Zhuoran Xu, Christopher Hardacre, Alex S. Walton, Andrew J. Logsdail, Sarah J. Haigh

**Affiliations:** † Department of Materials, University of Manchester, Oxford Road, Manchester M13 9PL, U.K.; ‡ Cardiff Catalysis Institute, School of Chemistry, Cardiff University, Park Place, Cardiff CF10 3AT, U.K.; § Department of Chemistry and Photon Science Institute, University of Manchester, Oxford Road, Manchester M13 9PL, U.K.; ∥ bp, Applied Sciences, Innovation & Engineering, Saltend, Hull HU12 8DS, U.K.; ⊥ bp, Applied Sciences, Innovation & Engineering, Chicago, Illinois 60606, United States; # Department of Chemical Engineering and Analytical Science, University of Manchester, Oxford Road, Manchester M13 9PL, U.K.

**Keywords:** Fischer−Tropsch, heterogeneous catalysis, cobalt, manganese, scanning transmission electron microscopy, *in situ*, promoters

## Abstract

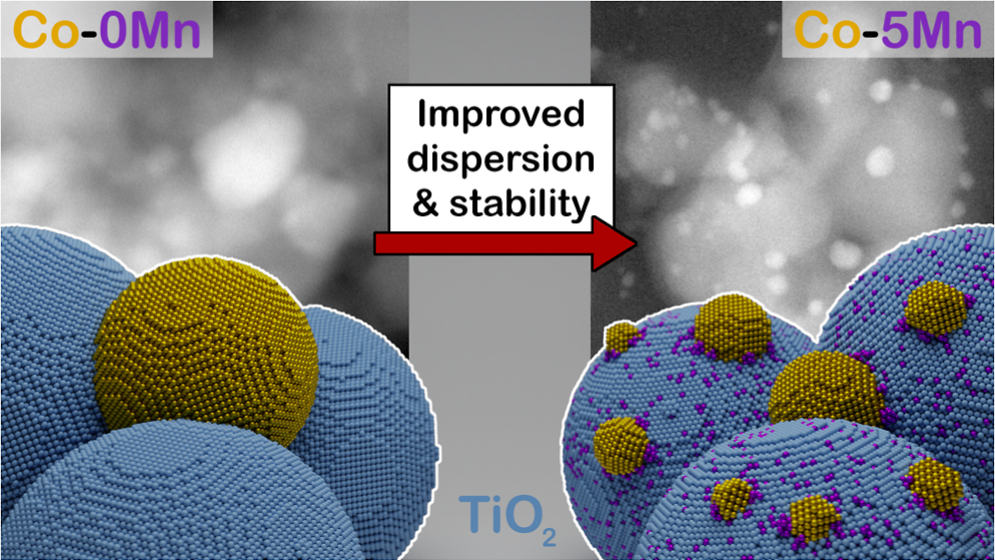

Modifying traditional Co/TiO_2_-based Fischer–Tropsch (FT) catalysts with Mn promoters induces a selectivity shift from long-chain paraffins toward commercially desirable alcohols and olefins. In this work, we use *in situ* gas cell scanning transmission electron microscopy (STEM) with energy-dispersive X-ray spectroscopy (EDS) elemental mapping, and near-ambient pressure X-ray photoelectron spectroscopy (NAP-XPS) to demonstrate how the elemental dispersion and chemical structure of the as-calcined materials evolve during the H_2_ activation heat treatment required for industrial CoMn/TiO_2_ FT catalysts. We find that Mn additions reduce both the mean Co particle diameter and the size distribution but that the Mn remains dispersed on the support after the activation step. Density functional theory calculations show that the slower surface diffusion of Mn is likely due to the lower number of energetically accessible sites for the Mn on the titania support and that favorable Co–Mn interactions likely cause greater dispersion and slower sintering of Co in the Mn-promoted catalyst. These mechanistic insights into how the introduction of Mn tunes the Co nanoparticle size can be applied to inform the design of future-supported nanoparticle catalysts for FT and other heterogeneous catalytic processes.

## Introduction

Fischer–Tropsch (FT) synthesis is a heterogeneous catalytic process that can convert syngas (CO/H_2_), typically derived from fossil fuels, into high-quality liquid hydrocarbon transport fuels.^1^ However, the environmental impact^2^ and energy security concerns^3^ of using fossil fuels are pushing the need for alternative, renewable sources of energy to meet with ever-increasing global demand.^4^ Recent technological developments have enabled the use of waste products, such as municipal solid waste, biomass, and natural gas, as an alternative feedstock, making FT synthesis a greener and more sustainable route to clean fuels and value-added chemicals.^5,6^

The traditional method for FT synthesis is to flow syngas over a metallic catalyst within a fixed-bed reactor.^7^ Several transition metals are active catalysts for FT, but the desire for high activity, selectivity, and stability makes cobalt-based catalysts the practical choice to produce high-molecular-weight paraffin fuels.^8^ Cobalt particle size is a key factor in FT performance, and diameters of 6 nm have been found to be optimal;^9^ larger particles have reduced surface area that leads to reduced activity, while smaller particles have a larger number of edge sites that favor chain termination and the formation of methane.^9,10^ In practice, it may be desirable to have a particle size below 6 nm in the starting activated catalyst if it provides longer onstream performance and if the selectivity for undesirable methane remains below an acceptable level, since particle size tends to increase during FT reaction conditions.^11^

Industrial FT processes rely on supported nanoparticle systems synthesized *via* wet impregnation, where the support is typically a metal oxide such as titania, silica, or alumina in the form of a nanoparticle powder.^12^ The support provides the catalytic material with mechanical integrity for the extruded pellets when loaded within the reactor as well as encouraging good dispersion and hindering metal particle agglomeration.^13^ The as-synthesized wet impregnated materials are then extruded into pellets, calcined at ∼300 °C in air for up to 12 h, and then subjected to a activation heat treatment inside the FT reactor (typically heating in hydrogen at 200–400 °C for 1–5 h) to produce the active metal phase of the catalyst.^14^ Metal–support interactions can also play a potential role in FT performance, such as modifying the charge transfer to create an interfacial layer with increased activity, reported previously for CO oxidation^15^ and CO_2_ hydrogenation;^16^ or through the use of reducible oxide supports, which form suboxide species during hydrogen spillover from the metal nanoparticle and that can migrate onto the metal catalyst surface,^17^ potentially enhancing the catalyst performance^18^ or blocking active sites and inhibiting chemisorption of CO and H_2_.^19^

The addition of alloying promoters, such as Mn,^20−22^ Re,^23−25^ or Zr,^26−28^ provides opportunities to tune the catalytic performance of the Co. For example, Re enhances the reducibility of cobalt, which produces energy savings by lowering the temperature required for catalyst activation^26,29^ and decreases the FT reaction selectivity to undesirable methane.^30^ The present study focuses on the use of Mn as a promoter, specifically industrial catalysts composed of wet impregnation-synthesized 10 wt % Co on TiO_2_ with and without 5 wt % Mn promoter. Previous investigations of 10 wt % Co on TiO_2_ have demonstrated that the addition of up to 10 wt % Mn as a promoter can modify the FT reaction selectivity toward long-chain oxygenates and liquid petroleum gas (LPG, C_2_–C_4_), with the addition of 5 wt % Mn identified as having unusually high selectivity toward desirable long-chain alcohols.^31,32^ The reason for the high selectivity of 10 wt % Co and 5 wt % Mn on TiO_2_ compared to that of the unpromoted catalyst is still debated despite the industrial importance of these materials for the FT reaction. Previous extended X-ray adsorption fine structure (EXAFS) analysis of the Co and Mn coordination in the catalysts has suggested a mixed metal oxide spinel (Co_*x*_Mn_3–*x*_O_4_) with a preference toward MnCo_2_O_4_ (*i.e.*, MnO·Co_2_O_3_) for the 5% Mn catalyst.^32^ The selectivity change on addition of Mn is thus suggested to result from the presence of MnO, which inhibits CO dissociation and instead favors CO insertion.^32^ Nonetheless, synthesis of catalysts using physical mixtures of Co-only and Mn-only catalysts on TiO_2_ have not shown similar catalytic behavior to those impregnated with a mixed solution, indicating that close interaction of Co–Mn is key to the differences in FT functionality.^31^ Similar EXAFS studies for γ-Al_2_O_3_-supported CoMn catalysts report that the presence of Mn leads to a stabilization effect of CO, C, H, O, and CH_*x*_ adsorption relative to unpromoted Co, in turn decreasing the CO dissociation barrier and increasing the intrinsic activity.^33^ However, Tucker *et al.* have used density functional theory (DFT) calculations to conclude that manganese promotion cannot be linked to enhanced CO adsorption or promotion of the C–O bond cleavage and that selectivity improvements toward liquid fuels are related to improved oxygen removal from the catalytically active surface.^22^

Both Co and Mn adatoms on anatase (101) have been studied previously with DFT. Yang and Zhou explored the binding sites of single Co adatoms on the surface as a part of the first-principles study with relevance to the hydrogen evolution reaction;^34^ similarly, Kalantari, Tran, and Blaha computed the stable configurations of clusters of metals and their oxides, including up to five atoms of Co and Mn.^35^ Previous computational studies have not, however, extensively considered coadsorption of Co and Mn adatoms as necessary to understand the current catalytic materials. Understanding the interplay of catalytic species is key to improving selectivity and long-term stability of the materials under FT conditions, but little is known about the nanoscale interactions of the active species in these materials. Spatially resolved analytical studies of how the material composition evolves during activation treatment have not been previously reported and could provide key new insights.

*In situ* transmission electron microscopy (TEM) is a powerful approach for imaging of supported nanoparticle catalysts,^36^ overcoming the limitations of traditional vacuum TEM studies by using ultrathin, electron-transparent silicon nitride (SiN_x_) membranes to separate the reactive gas environment from the microscope vacuum and using a local heating element to allow temperature control.^37,38^ Commercially available gas cell holders allow *in situ* TEM observations to be performed at pressures typically up to 1 bar and temperatures up to 1000 °C, which is highly desirable for studying the structure and composition of heterogeneous catalysts where the local environment and presence of surface adsorbates can strongly affect the surface chemistry and nanoparticle morphology.^39−41^*In situ* TEM is particularly valuable for reactive metal nanoparticle catalysts, like Co, where performing the activation of the catalyst inside the electron microscope prevents the exposure of the activated material to the atmosphere when transferring it to the transmission electron microscope.^42^ However, *in situ* TEM struggles to measure the local oxidation state of the materials due to the combined effects of the reactive gas environment and the amorphous SiN_*x*_ windows of commercial gas cells, producing significant electron scattering that degrades the quality of *in situ* electron energy loss spectroscopy (EELS) compared to *ex situ* studies. Complementary characterization using near-ambient pressure X-ray photoelectron spectroscopy (NAP-XPS) allows measurements of the changes in the cobalt oxidation state during reduction. Here, we apply a combination of *in situ* TEM and NAP-XPS to perform element-sensitive imaging and oxidation state analysis of wet impregnation-synthesized 10 wt % Co catalysts on titania with and without 5 wt % Mn (denoted Co-0Mn and Co-5Mn, respectively). We study these materials while heating in hydrogen across a range of elevated temperatures to reveal the evolution of the Co-rich nanoparticles during the catalyst activation step. DFT calculations are performed to understand the interactions of Co and Mn species with each other and with the titania support and thereby shed light on the mechanism for the observed decrease in nanoparticle size induced by Mn promotion.

## Results

### Catalytic Testing

**Figure 1:**
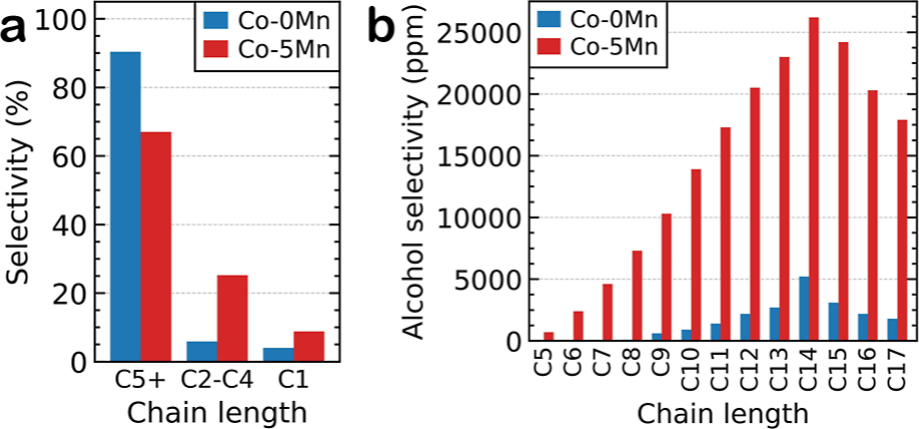
Catalytic testing of the 10 wt % Co/TiO_2_ catalyst with and without 5 wt % Mn. (a) The addition of Mn induces a selectivity shift from long-chain (C5+) products to those with shorter chains (C2–C4) as well as a slight increase in methane production. (b) The yield in alcohol selectivity for the C5+ products are significantly enhanced with Mn addition.

Catalytic testing was undertaken to investigate the conversion/product selectivity differences between the Co-0Mn and Co-5Mn catalysts^31,32^ (see Sections S1.1 and S1.2 for further details of the catalyst synthesis and reactor testing methods, respectively). The benefit of the Mn promotor is to enhance the selectivity to high-value alcohols and other oxygen containing products compared to the unpromoted Co-0Mn (Figure 1b). Unfortunately, this is only achieved at the expense of increased selectivity for undesirable C2–C4 products and methane (C1), as well as a slight overall drop of preferred C5+ products relative to Co-0Mn (Figure 1a). The 10% methane selectivity is slightly improved compared to the 18% reported for CoMn/TiO_2_ catalysts by Morales *et al.*,^43^ likely due to the larger particle size in our materials. However, Morales *et al.*(43) found the catalyst’s methane selectivity was not influenced by the addition of Mn, which we attribute to their use of a consecutive loading method of Co and Mn solutions *via* incipient wetness impregnation, as opposed to the simultaneous loading method (*via* mixed solution) employed for our investigations. This emphasizes the importance of understanding the interaction mechanisms for Co and Mn in tuning the catalytic performance, which is the focus of this study. A 0Co-5Mn catalyst previously analyzed by temperature-programmed reduction (TPR), presented in Figure S2, was also tested during the same reactor conditions, which showed practically no CO conversion and hence no catalytic activity toward FT synthesis (Table S1).

### Catalyst Dispersion and Chemical State in the As-Calcined Materials

**Figure 2:**
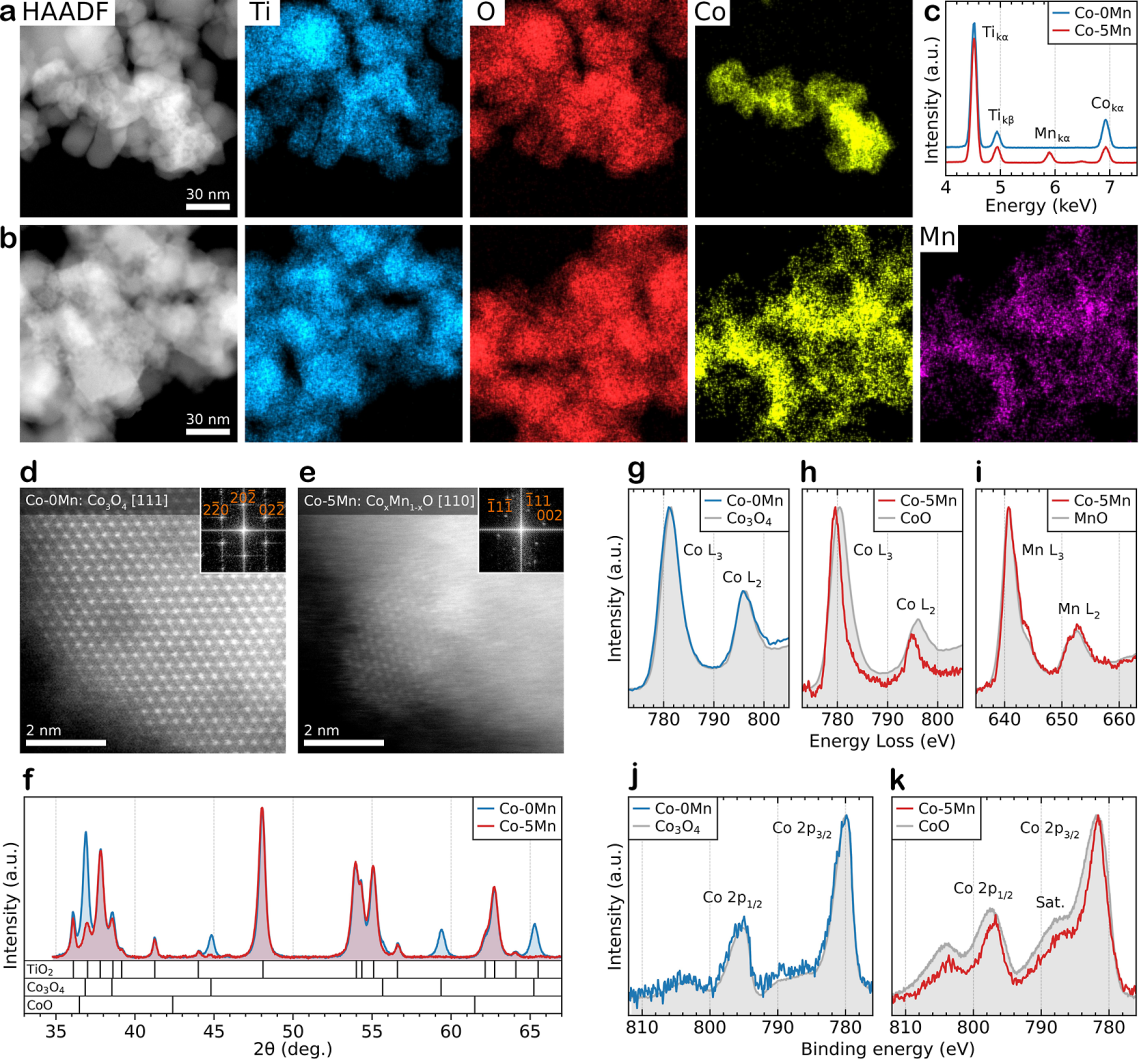
*Ex situ* structural and chemical characterization of the TiO_2_-supported Co/Mn catalysts after calcination (prior to activation). (a,b) HAADF-STEM imaging and STEM-EDS elemental mapping of the (a) Co-0Mn and (b) Co-5Mn catalysts. (c) Corresponding EDS spectra from the imaged regions in (a,b). (d,e) High-resolution HAADF-STEM imaging of the supported Co clusters in (d) Co-0Mn and (e) Co-5Mn. Fourier transforms of the images are shown as insets. Co-0Mn is indexed to the Co_3_O_4_ spinel viewed along [111], while Co-5Mn is indexed to CoO viewed along [110]. (f) XRD analysis highlighting the presence of Co_3_O_4_ in the Co-0Mn catalyst and the disappearance of any discernible cobalt oxide structure in the Co-5Mn catalyst. (g–i) Summed STEM-EELS edges from the (g) Co-0Mn and (h,i) Co-5Mn catalysts. (j,k) XPS spectra of the cobalt peaks from the (j) Co-0Mn and (k) Co-5Mn catalysts. The appropriate experimentally measured spectra taken from standard reference materials (Co_3_O_4_ spinel, CoO, metallic cobalt, Mn_3_O_4_ spinel, Mn_2_O_3_, MnO_2_, and MnO) are included in (g–k) for comparison.

High-angle annular dark field (HAADF) scanning TEM (STEM) images of the Co-0Mn and Co-5Mn catalysts after calcination, but prior to activation, are shown in the left-hand column of Figure 2a,b, respectively. These images reveal roughly spherical nanoparticles with diameters of 10–30 nm, in agreement with the expected particle size for the P25 titania support. For common scattering angles, the intensity of the HAADF-STEM signal scales with the local density, thickness, and atomic number, Z^1.7^.^44^ As the Co, Mn, and Ti oxides all have similar atomic numbers and density, but variable thickness, it is not possible to unambiguously distinguish the different oxide phases with only HAADF-STEM imaging. Energy-dispersive X-ray spectroscopy (EDS) elemental mapping and EELS were therefore required to characterize the dispersion of Co and Mn on the titania support.

The STEM-EDS elemental mapping of the as-calcined catalysts demonstrates the dramatic increase in the uniformity of Co dispersion when Mn is added (Figure 2a,b, with further examples in Figure S3). For the Co-0Mn catalyst, the Co forms irregular-shaped porous structures 20–100 nm in diameter (Figure 2a), which are much larger than the individual titania particles; in contrast, in the Co-5Mn catalyst, the Co is more uniformly dispersed across the support surface (Figure 2b). Comparing the elemental maps for Co and Mn in the Co-5Mn catalyst shows colocation of both elements, with high-resolution maps confirming that this is maintained at the nanoscale (Figure S4), consistent with previous studies of similarly prepared mixed-solution wet-impregnated catalysts at a lower spatial resolution.^31,32^Figure 2c shows the EDS spectra for both catalyst samples, confirming the presence of Mn only in Co-5Mn. A catalyst with 0 wt % Co and 1 wt % Mn (0Co-1Mn) on TiO_2_ was also prepared and demonstrated similarly high Mn dispersion to that of the Co-5Mn catalyst, confirming that Mn is likely the underlying cause of the increased Co dispersion effect (Figure S5).

Atomic-resolution HAADF-STEM imaging of the Co-rich regions in Co-0Mn and Co-5Mn (Figure 2d,e) shows that these also have different crystal structures, with Co-0Mn matching a Co_3_O_4_ spinel structure, while the smaller Co-5Mn particle closely matches the CoO/MnO rock salt phase; however, the lattice spacing of CoO and MnO are too similar to allow the particle composition to be distinguished using only HAADF-STEM imaging (Figure S6a,b). X-ray diffraction (XRD) analysis of the Co-0Mn catalyst confirms the Co_3_O_4_ spinel structure (Figure 2f); in contrast, no cobalt oxide (Co_3_O_4_ or CoO), manganese oxide (MnO, MnO_2_, Mn_2_O_3_, or Mn_3_O_4_), or mixed Co–Mn spinel (CoMn_2_O_4_ or MnCo_2_O_4_) phases are observed for the Co-5Mn material. The absence of crystallinity observed by XRD is likely due to the very small size of the crystallites or absence of long-range ordering; the particle in Figure 2e is ∼3 nm in diameter, and in other areas, the particle size was smaller or poorly defined, existing in a semicrystalline or amorphous state (Figure S6c) supported by the lack of Co/Mn peaks and increased background of the XRD profiles before subtraction (Figure S6d). Atomic-resolution HAADF-STEM imaging of the Co_3_O_4_ spinel in Co-0Mn suggests that the Co-rich particles have a crystallite diameter of the order of 10 nm. This is consistent with previous work on similar 10 wt % Co catalysts on titania, which also saw a reduction in the Co-containing oxide particle size in the promoted catalyst and where Scherrer analysis indicated a decrease in the average Co_3_O_4_ crystallite size from ∼11 nm when no Mn is present to ∼2 nm when 5 wt % Mn is incorporated.^31^

To establish the oxidation state of Co and Mn in the catalysts, STEM-EELS was used to obtain the Co and Mn L_2_ and L_3_ core-loss edges from the as-calcined catalysts (at energy losses of ∼780 and ∼640 eV, respectively), where these are then compared to Co and Mn oxide/metallic references (Figure 2g–i). For the Co-0Mn catalyst, the Co is a good match to the Co_3_O_4_ reference (Figure 2g), whereas for the Co-5Mn catalyst, the Co edges are best matched to CoO (Figure 2h) and the Mn edge is a good fit to MnO (Figure 2i). The change in the oxidation state of Co, from a mixed +3/+2 state (Co_3_O_4_) in Co-0Mn to being primarily +2 (CoO) in Co-5Mn, is also demonstrated by XPS analysis of the catalysts (Figure 2j,k) highlighted by the shakeup satellite in Co-5Mn at ∼787 eV, characteristic of Co^2+^.^45^ A full comparison of the catalyst Co and Mn STEM-EELS edges and Co XPS peaks to the full range of oxide/metallic reference materials can be found in Figure S7. Together, our HR-STEM, XRD, EELS, and XPS observations all suggest that, before activation, the Co and Mn species in Co-5Mn are dispersed across the surface of the titania support and are present as a disordered Co_1–*x*_Mn_*x*_O-type solid solution with some ultrafine-scale rock salt crystallites. This differs from the mixed spinel structure previously suggested for similar materials as the presence of CoO has only previously been reported for higher Mn promoter loadings and in Co catalysts made *via* a different synthesis route.^43,46^

### Evolution of the Catalyst Structure and Chemistry during *In Situ* Activation

**Figure 3:**
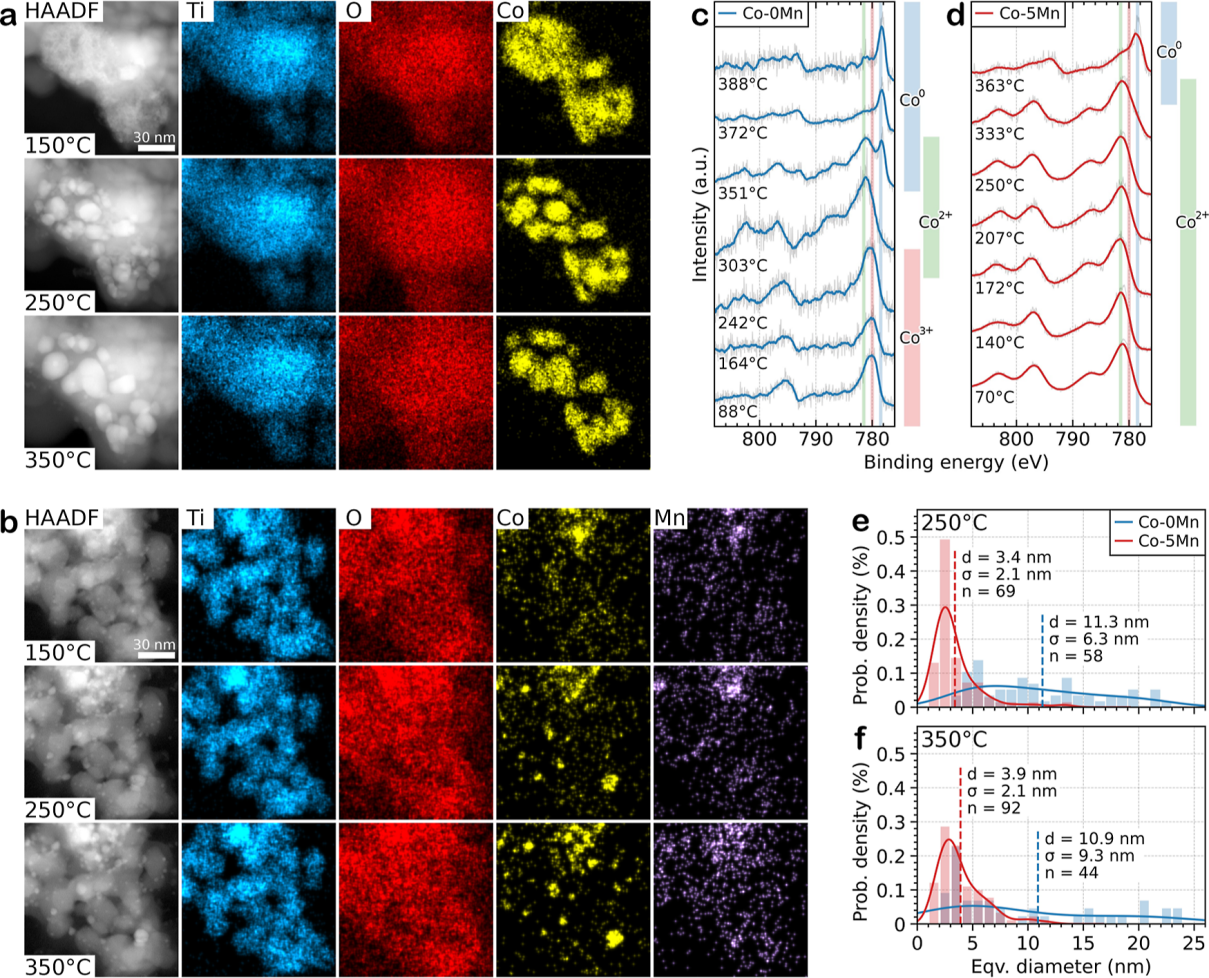
*In situ* structural and chemical characterization of the TiO_2_-supported Co/Mn catalyst dispersion during activation. (a,b) HAADF-STEM imaging and STEM-EDS elemental mapping of the (a) Co-0Mn and (b) Co-5Mn catalysts. Temperatures are shown inset on the corresponding HAADF images. (c,d) NAP-XPS analysis of the *in situ* activation of (c) Co-0Mn and (d) Co-5Mn in hydrogen. The presence of 5 wt % Mn induces a transition from a three-stage reduction to a two-stage process, where the presence of Co^3+^ indicates a spinel structure, Co_3_O_4_, which transitions to Co^2+^, indicative of CoO rock salt, in the promoted (as-calcined) catalyst and lowers the Co reduction temperature during activation. The reference data for the Co standards are presented in Figures 2i,j and S7d,e. (e,f) Histograms illustrating the Co particle size distributions for both catalysts at (e) 250 and (f) 350 °C (see Section S1.5 and Figure S16 for details).

We now seek to investigate the catalyst structure after the catalyst activation step, which is used to reduce the cobalt oxides to the metallic phase that is active for FT. HAADF-STEM and STEM-EDS/EELS investigations of the materials after an *ex situ* activation treatment (∼1 bar H_2_ at 300 °C for 12 h) revealed that Co-0Mn contains Co nanoparticles with a mean diameter of 17.3 nm (standard deviation, σ = 3.9 nm, and number of particles analyzed, *n* = 116, Figure S8a,c), consisting of metal cores and oxide shells (Figure S9c). The promoted Co-5Mn showed smaller particles, 11.4 nm in diameter (σ = 5.3 nm, *n* = 113, Figure S8b,d), with EELS providing a best fit to CoO and MnO (Figure S10). The high level of oxide is indicative of how readily Co can oxidize with minimal air exposure even though a vacuum transfer holder was employed to move the sample from the furnace to the transmission electron microscope (see Section S2.3 for STEM-EDS/EELS results and further details). Studying the nanoscale evolution of the catalyst structure and chemistry during activation therefore requires *in situ* techniques to be employed. *In situ* HAADF-STEM with EDS was performed using a Protochips Atmosphere gas cell holder, which has been designed to allow X-rays to reach the Titan scanning transmission electron microscope’s Super-X EDS detector geometry.^47^ The activation treatment was performed under ∼1 bar of hydrogen pressure at 150, 250, and 350 °C, with a 1 °C/s temperature ramp and a dwell time of 30 min at each temperature step, where the 150 °C starting temperature was chosen to avoid contaminant deposition prevalent during room temperature EDS (Figure S11). When the Co (or Mn) oxides transform to the metallic state during reduction, the increase in the local density of the material is visible as an increase in the HAADF-STEM intensity on the oxide support. Nonetheless, it is not possible to distinguish which metal species the reduced material contains, so STEM-EDS or EELS analysis is required. Unlike previous *ex situ* TEM characterization, using *in situ* STEM-EDS elemental mapping allowed us to investigate the evolution of the nanoscale dispersion of all constituent elements during the activation step (Figure 3, with EDS spectra presented in Figure S12), which has rarely been achieved. Tang *et al.* have recently applied *in situ* STEM-EDS elemental mapping to study a mixed Co and Pt system;^48^ however, this methodology has not previously been applied to CoMn/TiO_2_ catalysts, a system used industrially for FT synthesis.

STEM-EDS images of the Co-0Mn catalyst heated at 150 °C (Figure 3a) show a reduction of volume and porosity for the Co material compared to the room temperature morphology, although the Co-rich regions still have similar dimensions to those of the unreduced material (20–100 nm in diameter; for a comparison of *ex situ* and *in situ* imaging at room temperature, see Figures 2a and S13a, respectively). The sample was then heated to higher temperatures (250 °C and then 350 °C), causing the Co-rich regions to start sintering, forming distinct nanoparticles. To confirm that sintering was an effect of the activation treatment and not from the increased electron beam dose used during EDS, the regions in Figure 3 were imaged both before and after EDS acquisition at each temperature step (Figure S14). Particle size distributions were measured from the HAADF-STEM images using the Co EDS maps as a reference (maps are presented in Figures 3a and S15a, with a representative range of size analysis presented in Figure 3e,f and a complete range in Figure S16). The results reveal mean diameters of 11.3 nm at 250 °C and 10.9 nm at 350 °C, with standard deviations in the size distributions of 6.3 and 9.3 nm, respectively. Comparison of the same region at 250 and 350 °C shows several examples where individual particles have combined to a single particle, demonstrating that particle migration and coalescence occur during activation. The mean particle size does not increase despite the particle coalescence we observe. This is because new particles continue to form during activation, generating a bimodal particle size distribution; the peak particle size (mode) has decreased from 6.5 nm at 250 °C to 3.5 nm at 350 °C, but a second peak has also appeared at 20.5 nm after 350 °C activation. The number of particles (*n*) in the field of view has also decreased from 58 at 250 °C to 44 at 350 °C.

STEM-EDS images of the Co-5Mn catalyst activated at 150 °C in hydrogen show some clustering of the Co and Mn, although defined nanoparticles are not visible and both elements remain widely dispersed on the TiO_2_ (Figure 3b). After heating to 250 °C, distinct particles become visible in the STEM-EDS map for the cobalt distribution, and these become slightly larger and more clearly defined after further heating to 350 °C. Size analysis of the Co EDS maps reveals the mean diameter of these Co-rich particles to be 3.4 nm at 250 °C and 3.9 nm at 350 °C, with a standard deviation of 2.1 nm at both temperatures. More particles are visible for a region with the same field of view in Co-5Mn compared to that in Co-0Mn (for Co-5Mn, *n* = 69 at 250 °C and *n* = 92 at 350 °C), but care needs to be taken when comparing different samples as Co-0Mn is highly heterogeneous at the 100 nm length scale (Figure S3a).

Interestingly, comparison of the STEM-EDS mapping images for Mn and Co reveals a correlation between the two distributions at 150 °C, in agreement with our *ex situ* STEM-EDS measurements of the as-calcined sample. At 250 °C, there are two particles visible in the Mn map, both of which are close to particles that are visible in the Co elemental map. However, at 350 °C, the Mn is more uniformly dispersed than at 250 °C, and the Co and Mn distributions are no longer colocated but remain in close contact due to the high dispersion of Mn (in agreement with our STEM-EDS measurements after *ex situ* activation, as shown in Figure S8b). Together, these measurements show that the addition of 5 wt % Mn causes a significant decrease in the Co particle size and a narrowing of the particle size distribution in Co-5Mn compared to that in the unpromoted Co-0Mn catalyst. There is no evidence of alloying of Co and Mn in the particles formed at 350 °C. Previous studies have shown that Co particle growth can occur in supported catalysts through both particle migration and coalescence^11,49^ and *via* Ostwald ripening,^50,51^ and it seems likely that both also occur here in the Co-0Mn catalyst. Interestingly, no particle migration was observed in the Co-5Mn catalyst, although this cannot be ruled out to occur at higher temperatures.

*In situ* analysis of the local oxidation state of the Co and Mn in the catalyst particles during activation was not possible with STEM-EELS due to the poor signal-to-noise ratio in the summed spectra, which prevented the energy loss edge structure being distinguished for highly dispersed materials, such as Co in the Co-5Mn catalyst (Figure S17). Instead, NAP-XPS was used to measure the surface oxidation state of the catalysts as a function of temperature in hydrogen (Figure 3c,d). The NAP-XPS data demonstrate that the Co-0Mn material undergoes a transition from Co^3+^ to Co^2+^ between 242 and 303 °C and then from Co^2+^ to Co^0^ metal between 303 and 372 °C. In contrast, the Co-5Mn sample undergoes a single reduction from Co^2+^ to Co^0^ metal between 333 and 363 °C. The result is consistent with Co in the as-calcined Co-5Mn sample being present as CoO rock salt whereas the as-calcined Co-0Mn material is Co_3_O_4_. The result also demonstrates that at 250 °C, the Co-rich nanoparticles in the STEM-EDS, as shown in Figure 3a,b, are partially reduced for both Co-0Mn and Co-5Mn, whereas they are expected to be fully reduced at 350 °C. *In situ* XANES/EXAFS have also been used to confirm the predominantly metallic nature of Co in both catalysts postreduction in H_2_ at 300 °C for 8 h (Figure S18). Quantitative estimation of the oxidation state using linear combination fitting reveals that the contents of metallic cobalt in Co-0Mn and Co-5Mn were 98.3 and 96.8%, respectively (Table S2), suggesting that the presence of Mn acts to slightly inhibit the amount of Co that can be reduced. In the EXAFS, the Mn promotion also leads to a slight attenuation in the most intense peak at ∼2.2 Å, associated with scattering from the Co–Co bond, which can be explained by the smaller size of the Co particles (for further details, see Section S2.5).

### Theoretical Modeling of Co/Mn Interactions on the TiO_2_ Surface

To understand the two key observations of the Mn additions in (i) improving the distribution of Co in the calcined material and (ii) significantly decreasing the size of the Co-rich nanoparticles after reduction, DFT calculations were performed to determine the nature of the Co and Mn atomic interactions on the TiO_2_ support. Calculations considered metal atom coordination on the TiO_2_ anatase (101) surface, chosen due to the prevalence of the anatase TiO_2_ phase in our material and the high stability of this surface facet.^52,53^ An extensive range of adsorption complexes were considered for the Co and Mn species, with specific favorability observed toward the adsorption positions at various hollow and bridge sites between surface oxygen atoms.

The single-atom adsorption complexes for Co and Mn that result from our comprehensive configuration survey are shown in Figure S19 along with relative energies; atomic charges and preferred spin configurations are shown in Table S3. For both Co and Mn, positive atomic charge is observed, which indicates the favorability of oxidation states above zero. The most stable adatom configurations for both Co and Mn are with four nearest oxygen atoms, at distances of 1.87–4.12 Å for Mn and 1.81–3.06 Å for Co. For the Co adatom calculations, the five most stable adsorption positions have an energy range of 0.83 eV, with the two lowest energy Co adsorption complexes being notably close in geometry and energy, yet clearly distinguishable by oxygen bond lengths and distortion of the anatase lattice (Figure S19a,b); in contrast, for the Mn adsorption complexes, the four most stable structures have a large energy range over 1.80 eV, with the difference between the two most stable adsorption sites being 1.25 eV (Figure S19f,g). The much greater range of energies for Mn adatoms is indicative that they are anchored more strongly to the anatase (101) surface than Co and less likely to sinter at elevated temperatures. This is in excellent agreement with our STEM-EDS observations, where Mn was found to persist as a dispersed layer covering the titania support even after the Co has coalesced into clearly segregated nanoparticles. The most stable configuration of the Co and Mn adatoms have been previously identified as the hollow site between four oxygens,^34,35^ in agreement with our results in Figure S19a–c for Co and Figure S19f for Mn.

**Figure 4:**
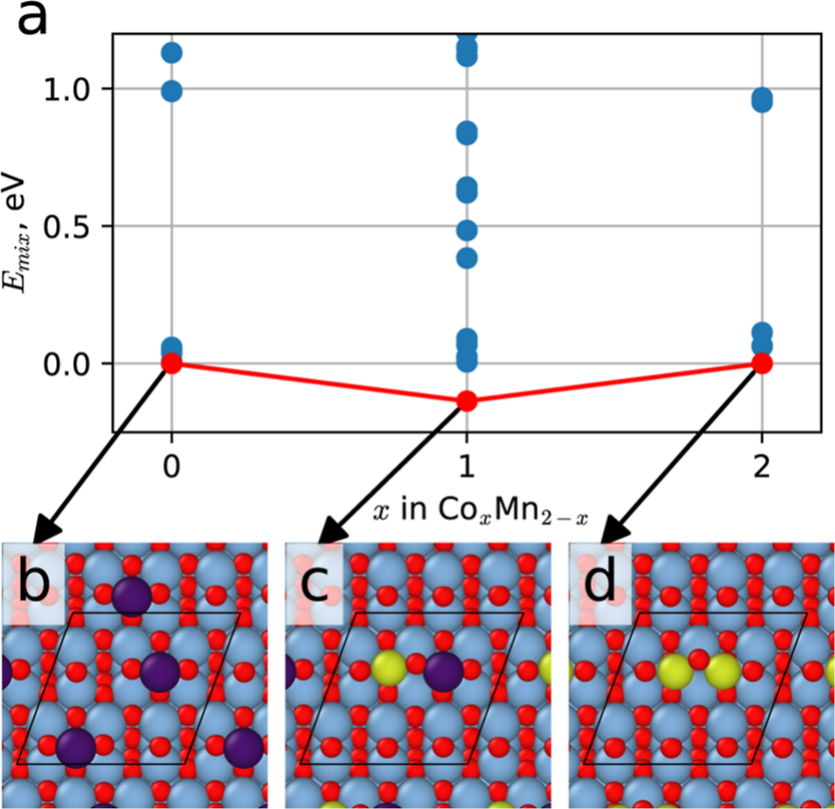
Most stable configurations for diatomic Co and Mn clusters. (a) Plot of the mixing energy, *E*_mix_ against diatomic composition for Co_*x*_Mn_2–*x*_ clusters. *E*_mix_ of the most stable mixed CoMn cluster is −0.14 eV, *i.e.*, more stable than the homonuclear diatomic clusters. The most stable configuration is indicated by the red dots, while other configurations are shown in blue. (b–d) Illustrations demonstrating the most stable configuration for (b) Mn_2_, (c) CoMn, and (d) Co_2_ on the anatase (101) TiO_2_ surface. Colors for Co, Mn, Ti, and O in the schematics are yellow, purple, blue, and red, respectively.

Diatomic adsorption complexes were further considered to understand the interactions between Co and Mn on the TiO_2_ anatase surface. The diatomic adsorption configurations considered favorability on available oxygen species at the TiO_2_ surface, guided by our observations for the monatomic adsorption (see Section S1.9 experimental method for details). Here, the chemical formula of the dimeric clusters is given as Co_*x*_Mn_2–*x*_, where *x* ranges from 0 to 2, and the relative energies of the configurations were considered by calculating the mixing energy, *E*_mix_, which is defined as
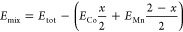
where *E*_tot_ is the total energy of the diatomic configuration, and *E*_Co_ and *E*_Mn_ correspond to the lowest energy configurations of the pure Co and Mn diatomic clusters, respectively. Figure 4a plots this mixing energy as a convex hull, with mixed heteronuclear diatomic clusters demonstrating energetic preference over homonuclear diatomic clusters. The most stable pairwise atomic configurations (with the lowest potential energy) are shown schematically in Figure 4b–d and demonstrate that Co–Co and Co–Mn interactions are readily formed for these species, while Mn–Mn interactions are unfavorable.

## Discussion

The DFT calculations provide important insights to explain the experimental changes in the catalyst morphology that we observe on addition of Mn to the Co-TiO_2_ catalysts. *In situ* and *ex situ* observations of the as-calcined catalysts reveal a dramatic increase in the dispersion of Co on the titania support in the presence of Mn and showed close mixing of Mn and Co to form CoO (and MnO) on the titania surface. Our DFT calculations show fewer stable lattice configurations for Mn than for Co, with large energy differences between metastable configurations, which suggests that Mn will be strongly bonded to the titania surface and cannot readily diffuse. As the most stable configuration for the CoMn clusters is found to be heteronuclear dimers, distributed Mn on the titania surface may act as anchors for Co species rather than favoring the nucleation of small homogeneous Mn clusters. These favored interactions are likely to lead to the experimentally observed improved Co dispersion on the TiO_2_ in the presence of Mn for the as-calcined material.

During the H_2_ activation treatment, STEM-EDS elemental mapping showed that Co sintered to form the active phase of cobalt metal nanoparticles, with the differences in the morphologies of the as-calcined material leading to a reduction in diameter from ∼11 to ∼4 nm through the addition of Mn, while the Mn remained dispersed over the support. Our DFT calculations also provide insights into the slowed coarsening of Co; the Co adatoms have several energetically accessible sites, which is likely to favor their diffusion across the surface to form Co-rich nanoparticles observed by STEM-EDS. Co–Co dimers are more stable than isolated Co adatoms on the titania, which indicates the favorability of nucleation for Co clusters. In contrast, Mn adatoms are constrained on the surface, with the next energy minimum 1.25 eV above the ground state, making surface migration of the Mn less likely than for Co; the Mn–Mn dimers are also unfavorable compared to the separated single Mn adatoms, which inhibits their agglomeration to form clusters and larger nanoparticles. These results may explain the persistent dispersion of Mn observed experimentally after the activation treatment.

The Co–Mn heterodimer pairs are found to be the most energetically preferable compared to any isolated adatom or homodimer species, which leads to a conclusion that the dispersed Mn adatoms on the TiO_2_ support act as surface anchoring points. During activation, these Mn adatoms provide multiple nucleation sites from which Co clusters can form, which together with the improved dispersion leads to the experimental observation of the active Co being present as smaller, more disperse nanoparticles. The Co particles are also more resistant to sintering in the Mn-promoted Co/TiO_2_ system as the Mn provides anchoring points on the titania, which slows surface diffusion of the Co and inhibits Ostwald ripening. Our STEM-EDS observations show that Mn remains distributed across the support after catalyst activation, so this effect will also likely continue to slow the sintering degradation of the catalyst during FT operation, helping to provide these industrial catalysts with a long lifetime on stream.

## Conclusions

In this work, we provide new insights to understand the effects that 5 wt % Mn addition has on the structure and chemistry of an industrial Co/TiO_2_ FT catalyst. The size of metal nanoparticle catalysts is known to be essential for optimizing the activity and selectivity of catalysts for FT as well as for other reactions in heterogeneous catalysis. Our complementary *in situ* analysis and DFT calculations illustrate how interactions at the atomic scale can improve the dispersion of wet impregnated elements on a substrate, reduce the particle size after activation, and inhibit the sintering of nanoparticle catalysts during operation. It is feasible that our observations of the Mn acting as the anchor site to disperse and inhibit the diffusion of the active catalyst element on the support could be expanded to provide small, sintering-resistant nanoparticles for other industrially relevant catalyst systems.

## Experimental Methods

Catalysts were prepared from mixed solutions of cobalt nitrate hexahydrate and manganese acetate tetrahydrate precursors dissolved in water at 40 °C. The solutions were incipient wetness coimpregnated onto a supporting P25 titania powder to yield 10 wt % Co + (*n*) wt % Mn/TiO_2_ catalysts, where *n* = 0 and 5. The resulting mixtures were extruded into pellets and calcined in air with a stepped heating profile (5 h at 60 °C, 5 h at 120 °C and 2 h at 300 °C). STEM with EDS and EELS was performed using a probe aberration-corrected FEI Titan G2 80–200 ChemiSTEM operated at 200 kV. *In situ* STEM analysis was performed using a Protochips Atmosphere gas cell system with H_2_ at pressures up to 1 bar and temperatures of 150–350 °C. Full details of STEM experimental measurements, DFT, TPR, XPS, and complementary X-ray absorption spectroscopy (XAS) methods are available in Section S1.

## Supporting Information Available

Experimental methods (catalyst synthesis/testing, TPR, STEM-EDS/EELS, XRD, XPS, XAS, and DFT) and additional results (STEM-EDS/EELS, XAS, and DFT) (PDF)
