# Supplementary material for: Tuning the Size of TiO2-Supported Co Nanoparticle Fischer–Tropsch Catalysts Using Mn Additions
Source: ACS Catal. 2024 Jul 1;14(14):10648–57. doi: 10.1021/acscatal.4c02721 (PMC11264206; doi:10.1021/acscatal.4c02721)
Supplement: Supplementary file 1 — cs4c02721_si_001.pdf [file cs4c02721_si_001.pdf]

Supporting Information

Tuning the size of TiO_2-supported Co nanoparticle Fischer-Tropsch catalysts using Mn additions

Matthew Lindley[1], Pavel Stishenko[2], James W. M. Crawley[2], Fred Tinkamanyire[3], Matthew Smith[1], James Paterson[4], Mark Peacock[4], Zhuoran Xu[5], Christopher Hardacre[6], Alex S. Walton[3*], Andrew J. Logsdail[2*], Sarah J. Haigh[1*]

[1] School of Materials, University of Manchester, Oxford Road, Manchester, M13 9PL, UK.

[2] Cardiff Catalysis Institute, School of Chemistry, Cardiff University, Park Place, Cardiff, CF10 3AT, UK.

[3] School of Chemistry, University of Manchester, Oxford Road, Manchester, M13 9PL, UK.

[4] bp, Applied Sciences, Innovation & Engineering, Saltend, Hull, HU12 8DS, UK.

[5] bp, Applied Sciences, Innovation & Engineering, Chicago, Illinois, 60606, USA.

[6] School of Chemical Engineering and Analytical Science, University of Manchester, Oxford Road, Manchester, M13 9PL, UK.

*sarah.haigh@manchester.ac.uk

*LogsdailA@cardiff.ac.uk

*alex.walton@manchester.ac.uk

Contents

1. Experimental Methods 3
  1.1. Catalyst Synthesis 3
  1.2. Catalyst Testing 3
  1.3. Temperature Programmed Reduction 3
  1.4. Transmission Electron Microscopy Sample Preparation 4
  1.5. Scanning Transmission Electron Microscopy 4
  1.6. X-ray Diffraction 6
  1.7. X-ray Photoelectron Spectroscopy 6
  1.8. X-ray Absorption Spectroscopy 6
  1.9. Density Functional Theory 7
2. Supplementary Results 9
  2.1. Catalyst Testing 9
  2.2. Ex situ STEM of the catalyst dispersion prior to activation 10
  2.3. Ex situ STEM of the catalyst structure and chemistry after activation 13
  2.4. In situ STEM of the catalyst morphology change during activation 18
  2.5. X-ray Absorption Spectroscopy 23
  2.6. Density Functional Theory 25
3. References 26

Section 1: Experimental Methods

S1.1 Catalyst Synthesis: Catalysts were prepared from precursor mixtures containing 627g of cobalt nitrate hexahydrate (Alfa Aesar, ACS 98-102%, code: 36418), with the promoted catalyst also containing 54g of manganese acetate tetrahydrate (Sigma Aldrich, >99%, code: 221007), each dissolved in 150 ml DI water at 40°C. The solution was incipient wetness co-impregnated onto a supporting titania (Evonik P25 aeroxide – 1 kg) powder and mixed thoroughly to create a paste. No binders or peptising agents were used. The precursors were added in quantities to target metal loadings of 10wt% Co in both catalysts, with the promoted catalyst target of 5wt% Mn. The paste was added to a Bonnet single screw extruder to form trilobe shaped pellets (1.6 mm diameter). The extrudates were then dried in air (5 hrs at 60°C, 5 hrs at 120°C) and then calcined in air (2 hrs at 300°C) in a fan assisted box furnace to control the precursor decomposition and the release of water from the catalyst.

S1.2 Catalyst Testing reactor testing was carried out in a 16-fold fixed bed, high throughput micro reactor using 1.0 mL of catalyst and used common gas feeds/pressures, with the ability for independent temperature control of each bed. Catalyst activation to reduce the cobalt oxide to the active metallic phase was conducted within the reactor using 100% H_2 at 300°C. Catalysis test conditions used a syngas ratio of 1.8 H_2:CO, 30 bar pressure and gas hourly space velocity (GHSV) of 1500 hr^{-1}, where both catalysts were screened simultaneously. The temperature of the Co-0Mn and Co-5Mn catalysts were individually controlled to maintain similar CO conversion throughout the reactor testing, where an online internal standard GC method provided conversion and selectivity measurements up to C17.

S1.3 Temperature Programmed Reduction (TPR): TPR analysis was conducted using a Quantachrome ChemBET Pulsar chemisorption analyzer. 100 mg of fresh calcined catalyst was loaded and dried under flowing argon, heating from room temperature at a rate of 5°C/min to 110°C and dwelling for 15 min. The sample was then allowed to cool to room temperature before the reduction treatment, where gas flow was changed to 100% H_2 (60 mL/min) and temperature increased to 600°C (5°C/min). Once at temperature, the sample was left to dwell for 30 mins to

equilibrate and allow for full reduction of the cobalt oxide. H_2 content in the gas outlet was measured throughout the reduction procedure using an integrated thermal conductivity detector.

S1.4 Transmission Electron Microscopy (TEM) Sample Preparation: For TEM observation, the calcined catalyst pellets were ground to a fine powder and dispersed in methanol solution for drop casting onto the appropriate TEM support. For ex situ analysis, the samples were deposited onto holey carbon copper or gold grids (Agar Scientific) and baked in vacuum at 100°C for 3 hours to aid in desorption of carbonaceous species, which may lead to contamination build up during electron beam exposure. Ex situ activation of the catalysts on the as deposited grids was performed in a Carbolite tube furnace, heating from room temperature at a rate of 5°C/min to 300°C with pure H_2 gas flowed at 50 ml/min. Observations at 3 hrs activation and 12 hrs activation were made to investigate catalyst morphology and elemental dispersion. To minimise oxidation during transfer of the sample from the furnace to the TEM the sample was sealed in the tubing during H_2 flow, left to cool and then transferred to a glove box environment (Argon pressure 1 bar, < 500 ppm oxygen), where it was subsequently loaded into a Fischione Model 2560 vacuum-transfer TEM holder. For in situ analysis, the sample was dropcast onto the 30 nm thick SiN_x window of Protochips Atmosphere gas cell top chips.

S1.5 Scanning Transmission Electron Microscopy (STEM): STEM imaging and complementary analytical techniques were performed using an aberration-corrected FEI Titan G2 80-200 ChemiSTEM, located at the University of Manchester, operating at 200 keV and a beam current of 90 pA. This microscope is equipped with an FEI Super Quad Silicon Drift Detector with a total collection angle of ≈0.7 sr, from which energy dispersive X-ray spectroscopy (EDS) data was acquired using the Bruker Esprit software package. High-angle annular dark field (HAADF) STEM imaging was performed using FEI TIA and Gatan DigitalMicrograph software. Electron energy loss spectroscopy was acquired using a GIF Quantum ER System (Gatan Inc.) with 0.1-0.5 eV/channel dispersion and dwell times of 0.1-0.2s to attain the core-loss acquisitions. The electron beam dose used during ex situ analysis of the catalysts was chosen to avoid any significant visible changes to the samples by beam damage (beam fluence of 3.4×10^6 e/nm^2 and flux of 1.6×10^5). During in situ analysis the total allowable beam fluence was reduced to 3.4×10^5 e/nm^2) to avoid the potential for faster beam damage during exposure to activation conditions. Observations of the

regions of interest before and after EDS acquisition (Figure S14) showed no significant change occurred as a result of the electron beam). Post-processing and visualisation of STEM HAADF images, EDS and EELS datasets was performed using Python-based libraries (Numpy[1], Scipy[2] and Matplotlib[3]).

Particle size analysis was performed on the acquired HAADF STEM images using ImageJ to select the projected 2D area of each particle, with EDS elemental mapping used to discern the Co particles from the TiO_2 support. The measured areas were then recalculated as equivalent spherical areas to give a representative diameter. Due to the difference in height of the particles on the support (i.e., along the beam direction) many had edges which were not in focus, making area selection subjective. To address error in repeatability, the measurement process was undertaken three times, with the average value of these measurements used in calculating the size histograms. The measurement error was found to be small compared to the standard deviation in the measurements so was considered negligible.

In situ gas cell STEM EDS/EELS measurements were performed using the Protochips Atmosphere system on the Titan. This involved the construction of two Si MEMS-fabricated chips, each containing an electron transparent SiN_x membrane window, into a low penumbra geometry holder. The top chip provides pre-calibrated heating to the sample, up to 1000 °C, via electrical contacts and is temperature controlled using proprietary software. The bottom chip has a 50 nm membrane and spacers which sit against the top chip to provide a separation through which gas can flow at up to 1 bar pressure. After holder insertion into the microscope, but prior to any activation treatments used, the closed cell (with sample contained within) was pump/purged with pure argon (N6.0, ≤0.5 ppm oxygen) several times to aid removal any contaminants (e.g., oxygen and water) which may hinder imaging and/or affect the activation process.

To study the catalyst response to the activation treatment, the in situ gas cell system was filled with 1 bar H_2 and the sample heated from room temperature to 150, 250 and 350°C. Research grade hydrogen (N5.5, ≤0.5 ppm oxygen) was used to maintain the lowest levels of contamination possible. A relatively fast heating rate of 1°C/s was used to minimize the changes during the heating ramp. This is achievable with the small sample size in an in situ cell but we note that slower heating was used for the ex situ bulk investigations. A dwell time of ~30 minutes at each temperature increase was implemented to allow the system to equilibrate before observation and at each stage the same regions of interest were then subjected to a series of HAADF-STEM and

STEM-EDS data acquisitions. STEM-EDS and STEM-EELS data acquisition was avoided at low temperature (less than 200°C), as this is known to result in electron beam induced contamination which could affect the later reduction of the catalyst. Figure S8 demonstrates this occurrence after STEM-EDS mapping for 2 minutes at room temperature.

S1.6 X-ray Diffraction (XRD): XRD was collected with Cu K_{α} radiation (8.04 keV) using a Bruker XE-T detector. The angular range collected was 10-70° 2θ, with a 0.02° step size and dwell time of 8 seconds per step. Data collected from the catalysts were compared to reference peaks for TiO_2 (ICDD PDF codes for rutile: 00-021-1276, anatase: 00-021-1272), Co_3O_4 spinel (PDF code: 00-042-1467) and CoO (PDF code: 00-048-1719).

S1.7 X-ray Photoelectron Spectroscopy (XPS): All XPS spectra were recorded with a SPECS NAP-XPS system employing a monochromatic Al Kα source (1486.6 eV) and a 'Devi-Sim' cell-type NAP environment attached to a SPECS Phoibos 150 NAP differentially pumped analyser. For standard XPS analysis (Figure 2), the catalyst powders were pressed onto carbon tape. For the in-situ reduction NAP-XPS measurements (Figure 3), the catalyst powder was hammered into a titanium foil substrate. The spectra were recorded at a pass energy of 30 eV and charge corrected using the Ti 2p peak taking 459.3 eV as the reference value for TiO_2. For the in-situ reduction experiment 1 mbar H_2 was admitted to the NAP cell and spectra acquired for all the temperatures specified in Figure 3. The acquisition time for XPS spectra at each temperature was about 6 h.

S1.8 X-ray Absorption Spectroscopy (XAS): In situ XAS was employed to confirm the catalyst sample had fully reduced under similar conditions to be used for in situ STEM observations (pure H_2 flow at 350°C). XAS sample preparation involved mixing 5.0 mg of each catalyst powder with 25.0 mg of BN and pressing the mixtures into a pellet. Catalyst samples were packed into a six-shooter situated in a sealed in-situ sample cell. 100% H_2 was fed into the sample cell at 100 mL/min. The temperature was then increased at a rate of 5°C/min to 300°C, where it was then held for 8 hr. The cell was then cooled down to room temperature to acquire XANES measurements. For EXAFS measurements, the same sample cell was re-heated, again in pure H_2, at a rate of 5°C/min to 350°C, where it was then held for 1 hr. The cell was then cooled again to room temperature for measurements to be taken.

S1.9 Density Functional Theory (DFT): Catalyst modelling calculations were performed using DFT as implemented in the FHI-aims software package[4]. The unpaired ground state electronic configuration of Co and Mn elements required application collinear spin calculations, with spin restrictions enforced where discussed. Relativistic effects were included via the zero-order regular approximation (ZORA)[5]. Due to an excellent ability to reproduce the bulk and surface properties of TiO_2, and design around accurate surface adsorption chemistry, the mBEEF exchange-correlation density functional was used[6]. The standard *light* basis sets (2010 version), as distributed with FHI-aims, were used to represent the electronic wavefunction. Convergence of the self-consistent field (SCF) cycle, unless stated otherwise, was deemed complete when the total energy changed by less than 10^{-6} eV between iterations.

For the surface geometries of anatase (101) TiO_2, it was necessary to create a supercell with surface dimensions of 3x2 unit cells, and depth of 2 unit cells (i.e., 4 Ti layers). A converged **k**-grid of 3x3x1 was applied for all calculations therein, with no sampling in the z-direction due to the finite nature of the slab model. For subsequent geometry optimisations, the bottom unit cell layer of the slab model was fixed, and a dipole correction included to prevent spurious electrostatic effects from applying a one-sided model. Geometry optimisations were deemed complete when the maximum force on any atom was below a threshold of 0.01 eV/Å.

To understand the interaction of Co and Mn atoms with the anatase (101) surface, we have identified adsorption complexes constituted by these adatoms. As bonding to O species is preferred for metal atom adsorption, a set of adsorption complexes was generated by placing metal adatoms on top, on bridges, and in hollows between surface oxygen atoms. There are three unique surface oxygen atoms on the anatase (101) surface unit cell (Figure S1). 12 unique sites were selected (3 atop, 6 bridges, 3 hollows). Therefore, 24 initial configurations were built (12 sites for each metal). To build initial configurations of two-atom clusters only atop sites were used to avoid trapping in local energy minima. All pairwise configurations with distance between adsorbate atoms up to 6 Å were enumerated. Then, after first geometry optimization stage, all homotopic combinations of each optimized cluster were added to the pool of clusters. For example, for each Co-Co cluster we added Co-Mn, Mn-Co, and Mn-Mn clusters with the same atomic coordinates. Subsequently, the geometry optimization was performed on the extended pool of clusters. The SuSMoST package was employed for enumeration of single-atom adsorption sites as well as two-atom clusters, considering only symmetry inequivalent configurations to save computational resources[7].

To avoid adatoms becoming trapped at saddle points during optimisation, minor random displacements were introduced using the ASE (Atomic Simulation Environment) rattle functionality. Due to challenges with convergence of the SCF cycles, and to minimise computational cost, a multi-step approach was then taken to geometry optimisation: initially coarse relaxation and DFT settings. Firstly, an optimisation was performed with the PBE exchange-correlation density functional[8], using a spin-paired configuration and coarse convergence criteria of changes in density below 5 x 10^{-4} e for the SCF cycle, and forces below 0.05 eV/ Å for geometry optimisation; this allowed identification of the possible stable positions of the metal adatoms, from which structures were further optimised with higher accuracy, spin-unpaired approach as highlighted above. The free atom's unpaired spin configurations were used as the initial starting point for each structure: 3 for Co, and 5 for Mn.

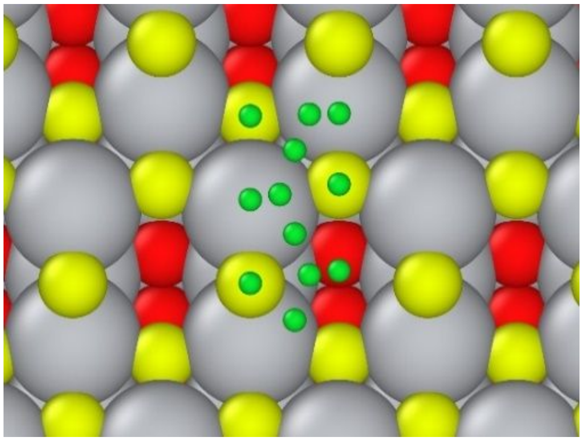

Figure S1: Surface oxygen atoms (yellow) on anatase (101) surface. Small green spheres show adsorption sites for metal ions. Other oxygen atoms are red and titanium atoms are grey.

Section 2: Supplementary Results

S2.1 Catalyst testing

Temperature programmed reduction (TPR) was used to investigate the bulk reduction behaviour of the Co-0Mn and Co-5Mn catalysts during the activation treatment (Figure S2). A typical two-stage activation is suggested in Co-0Mn, where the peaks R_1 and R_2 are associated with the reduction of Co_3O_4 to CoO and CoO to Co^0 (metal), respectively. A relative decrease in peak temperatures can be observed in the promoted catalyst, with the reduction to Co^0 in the Co-5Mn catalyst ~70°C lower than in Co-0Mn. This raises questions over the origin of the low temperature peak, particularly when considered together with the NAP-XPS data that demonstrates a single stage reduction in Co-5Mn. Low temperature features may potentially arise from the reduction of residual cobalt nitrates and precursor salt remaining from the calcination process. A shoulder peak in R_2 was also seen around 300°C in the Co-0Mn catalyst, which is often attributed to the reduction of cobalt titanate species[9,10]. This peak appears to have decreased in the Co-5Mn catalyst profile, although this may be obscured due to the shifting of the more intense CoO to Co^0 peak to lower temperatures. A 5wt% Mn/TiO_2 catalyst containing no Co (0Co-5Mn) was also subjected to the same TPR conditions from which no peaks were observed, revealing all transitions concerned arise from cobalt reduction as opposed to reduction of any Mn oxide species.

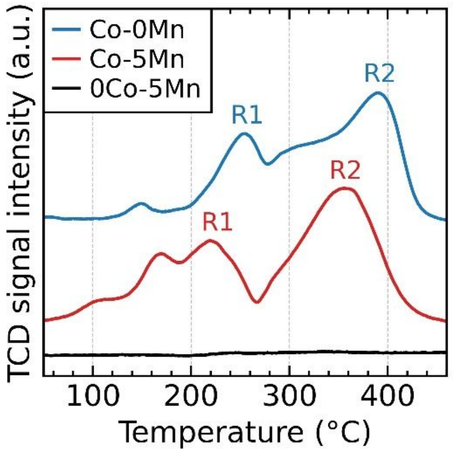

Figure: S2: TPR results suggesting a typical two-stage reduction from Co_3O_4 to CoO (R1) and eventually to Co metal (R2) in both catalysts, with the Co-5Mn catalyst showing a decrease in temperature required for these transitions. In contrast, a catalyst with no Co and 5wt% Mn/TiO_2 shows no reduction peaks.

Catalyst	CO conversion (%)	Selectivity (%)		
		C5+	C2-C4	C1
Co-0Mn	59.7	90.4	5.9	4.0
Co-5Mn	62.3	67.0	25.2	8.8
0Co-5Mn	0.5	-	-	-

Table S1: CO conversion of the tested catalysts showing a slightly increased CO conversion and significantly decreased C5+ selectivity for the Co-5Mn catalyst relative to Co-0Mn. 0Co-5Mn show no significant CO conversion occurring.

S2.2 Ex situ STEM of the catalyst dispersion prior to activation

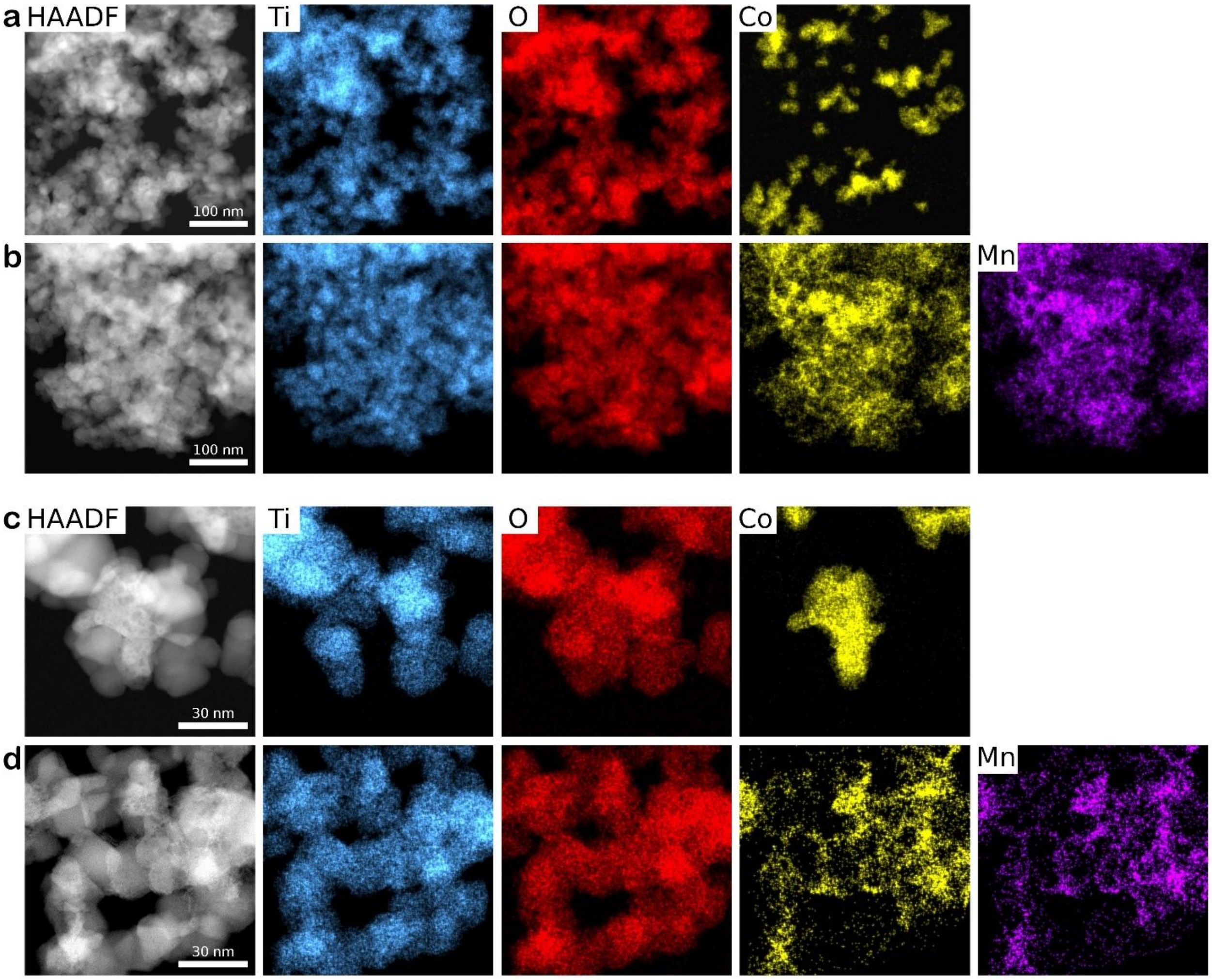

Figure S3: HAADF-STEM imaging and corresponding STEM-EDS elemental mapping of the as-calcined CoMn/TiO_2 catalysts. (**a,b**) Low and (**c,d**) medium resolution STEM images of the (**a,c**) Co-0Mn and (**b,d**) Co-5Mn catalysts demonstrate the increased distribution of Co as a result of Mn addition. (**e**) EDS spectra acquired from the regions mapped in (a) and (b).

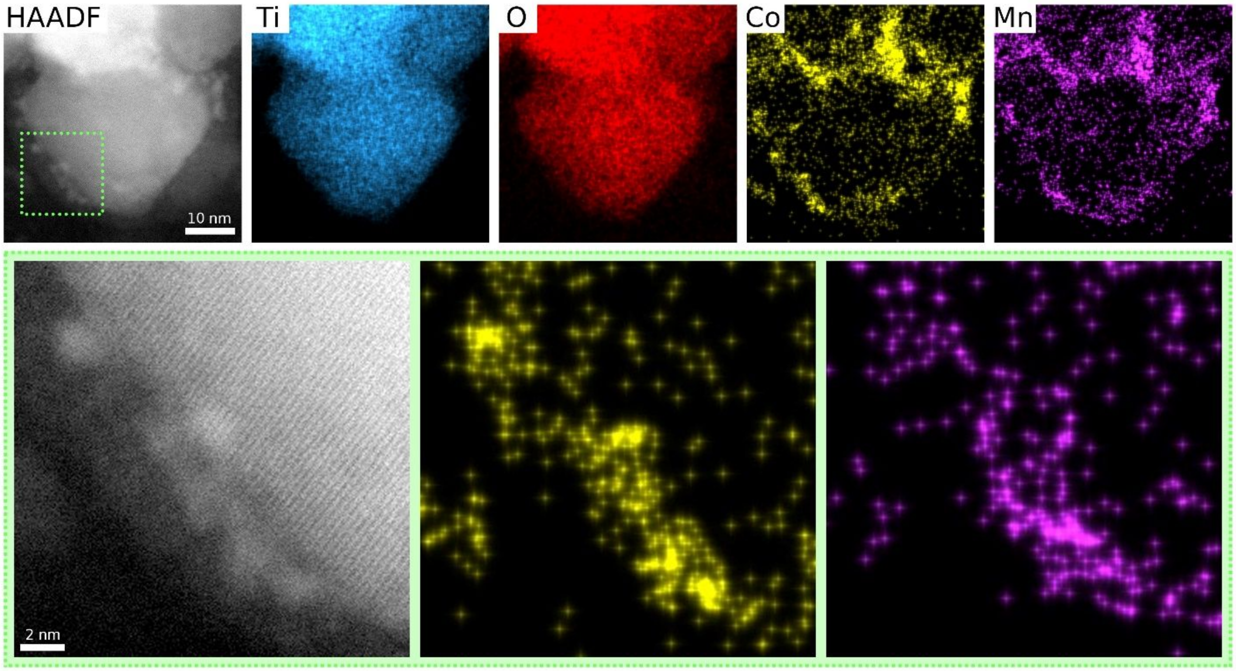

Figure S4: HAADF-STEM imaging and corresponding STEM-EDS elemental mapping of the as-calcined Co-5Mn catalyst. Colocation of the Co and Mn from high resolution STEM imaging/mapping demonstrates the formation of a mixed material at the atomic scale.

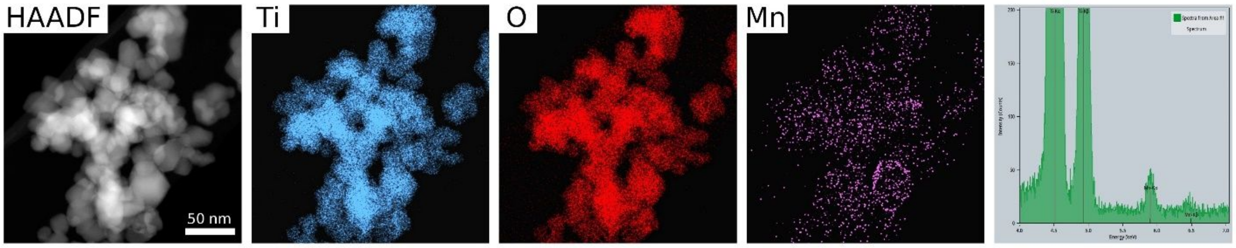

Figure S5: HAADF-STEM imaging and corresponding STEM-EDS elemental mapping of the as-calcined 0Co-1Mn catalyst. The maps demonstrate a high dispersion of Mn across the TiO_2 support is preferential without Co present, consistent with previous observations of high dispersion with Co addition.

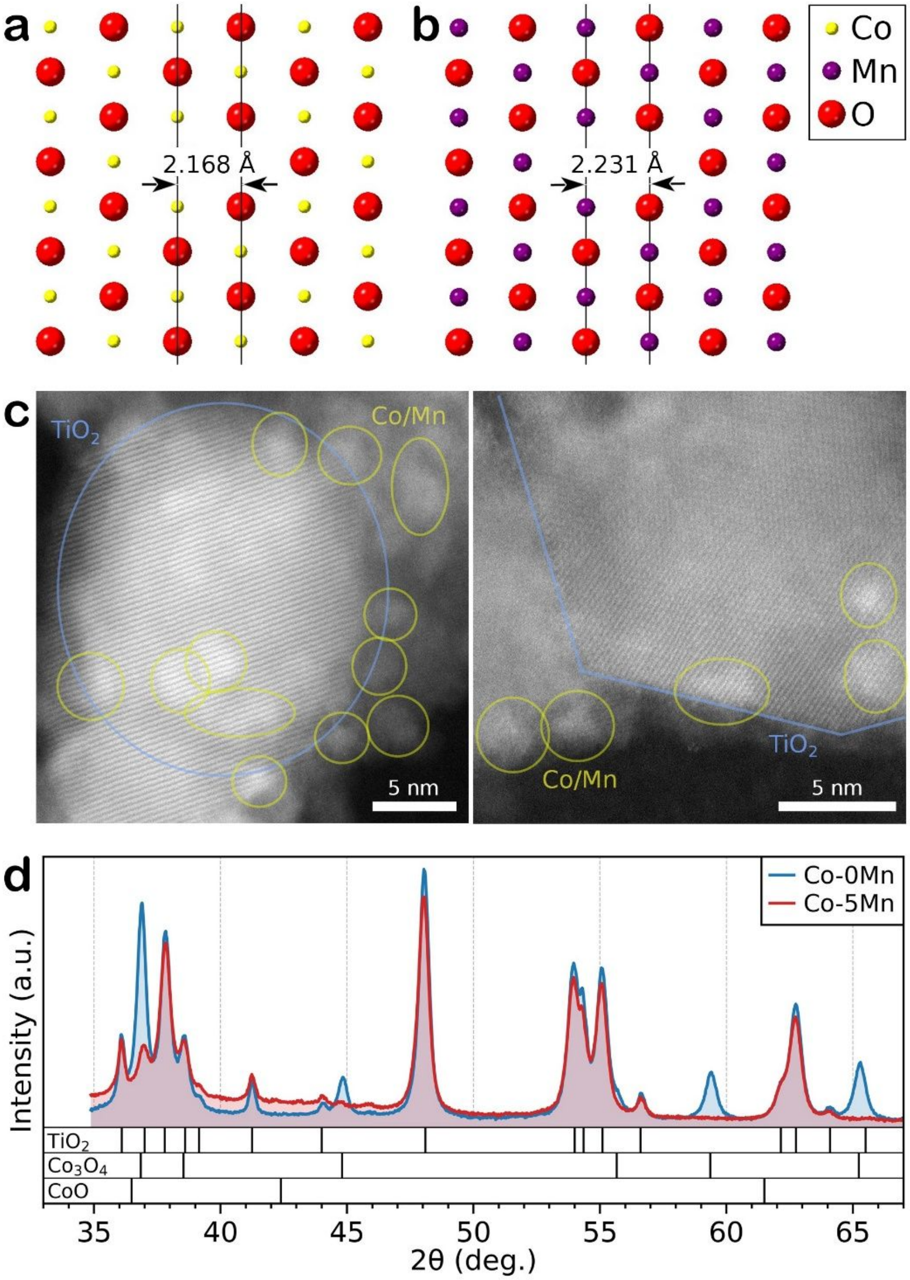

Figure S6: High resolution HAADF-STEM imaging of the as-calcined Co-5Mn catalyst. (**a,b**) Rocksalt crystal structure of (a) CoO and (b) MnO oriented along the [111] direction highlighting the similarity of (001) lattice spacings. (**c**) The presence of small, mostly disordered Co/Mn nanoparticles on the TiO_2 surface explains the absence of crystallinity observed by XRD in (d). (**d**) XRD profiles showing the relatively high background (pre-subtraction) of Co-5Mn compared to Co-0Mn, suggesting an increased amount of amorphous material in the catalyst.

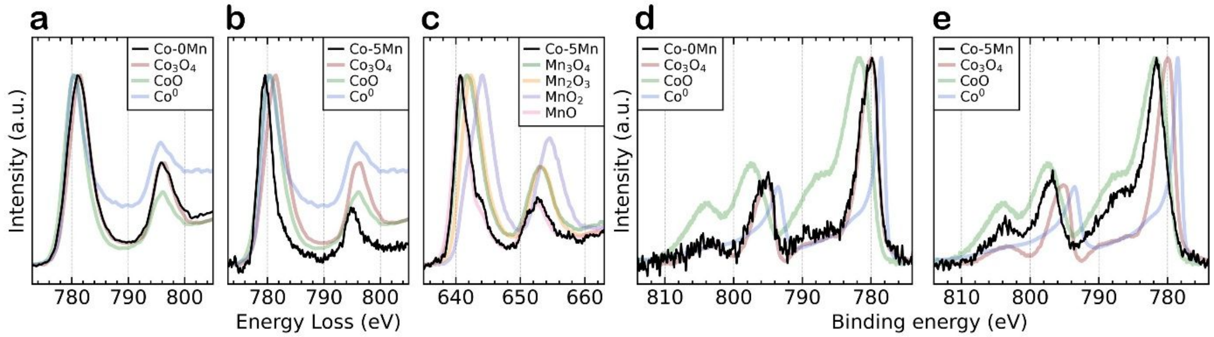

Figure S7: Structural analysis of the as-calcined CoMn/TiO_2 catalysts via STEM-EELS, XPS and XRD. (**a-c**) STEM-EELS of (a) Co edges acquired from the Co-0Mn catalyst, (b) Co edges and (c) Mn edges acquired from the Co-5Mn catalyst. Results show the change of Co structure from Co_3O_4 to CoO with addition of Mn, which takes the form of MnO. (**d,e**) XPS of the Co peaks acquired from (d) the Co-0Mn catalyst and (e) the Co-5Mn catalyst, showing consistent results to that of STEM-EELS.

S2.3 Ex situ STEM of the catalyst structure and chemistry after activation

Ex situ STEM EDS and EELS was applied to investigate the morphological change of the FT catalysts after an industrially essential activation step required to reduce the cobalt to its catalytically active metal state. Specifically, a reduction treatment in flowing pure H_2 gas (50 ml/min) at 300°C (5°C/min ramp) was performed in a furnace and the sample was then transferred to a Fischione Model 2560 vacuum-transfer TEM holder in a glove box to minimise air exposure prior to insertion into the TEM vacuum. STEM EDS elemental mapping of the Co-0Mn (unpromoted) catalyst reduced for 3 hrs show Co clusters that still contain remnants of the porous features seen in the fresh calcined sample (Figure S3a,b), whereas reduction for 12 hrs leads to complete densification of the Co to form particles with approximately spherical geometry (Figure S3c) and a mean diameter of ~17 nm (Figure S4a,c). For the Co-5Mn catalyst, the 12 hrs reduction treatment results in smaller Co nanoparticles, with a mean diameter of ~11 nm (Figure S4b,d). In contrast to the pre-reduced catalyst, the Mn distribution after reduction does not correlate with the Co distribution, with the Mn remaining uniformly dispersed on the TiO_2 support. Ex situ STEM-EELS of the reduced catalysts shows that for Co-0Mn, Co_3O_4 has reduced to metallic Co (Figure S5a,b) while for Co-5Mn, the Co edges still fit best to CoO (Figure S5c,d) similar to the as calcined catalyst. The Mn edges in Co-5Mn also remain closely matched to the reference for MnO both before and after reduction.

The accuracy of this particle size, chemical and elemental analysis from ex situ analysis is questionable since a detailed comparison of the HAADF-STEM image, and of the Co and O STEM-EDS maps after the 12 hrs reduction for Co-0Mn (Figure S3c) reveals that the reduced Co particles display a core-shell structure; where the outer shell is associated with a ~3 nm thick oxygen rich layer. This observation of an outer shell with an increased oxygen content relative to the core suggests that despite efforts to minimise air exposure, the Co particle surface oxidised after the furnace reduction. The edges of the catalytic nanoparticles are also poorly defined for both Co-0Mn and Co-5Mn, which also suggests surface oxidation which will be likely to have artificially increased the particle size. This oxidation has also likely changed the local oxidation state which prevents accurate chemical analysis being extracted from the STEM-EELS. The smaller size of the nanoparticles in the Co-5Mn catalyst means that exposure to oxygen during sample transfer is likely to have had a larger negative effect on the accuracy of nanoparticle size and overall oxidation state measurements. This motivates the use of in situ gas cell STEM elemental analysis to investigate evolution of the Co nanoparticles during the catalyst reduction.

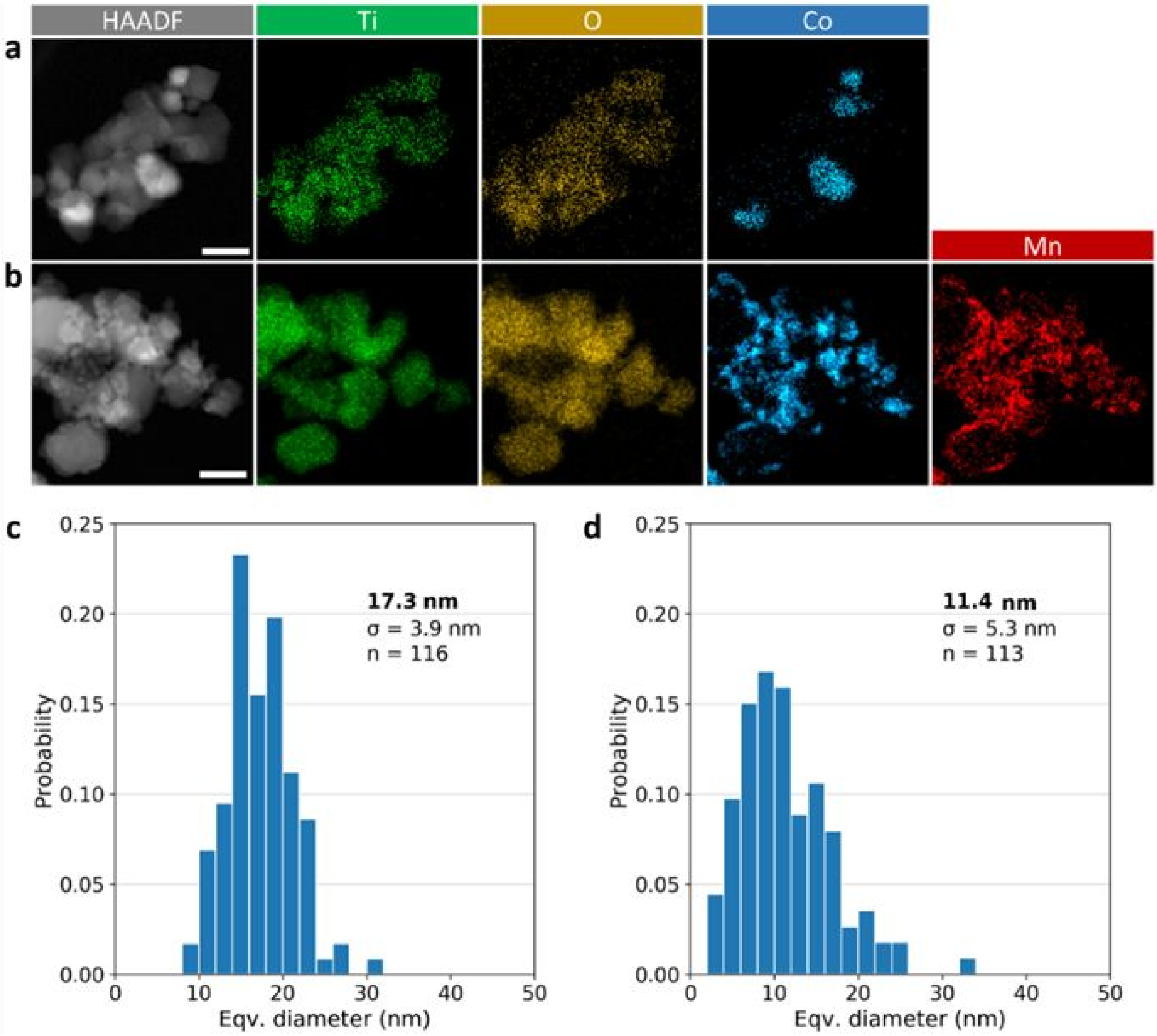

Figure S8: (a,b) STEM-EDS elemental mapping to compare the Co (and Mn) distribution after ex situ activation for 12 hours in 1 bar pure H_2 at 300°C. (**a**) Co-0Mn (10 wt% Co/TiO_2 catalyst without Mn promotion). (**b**) Co-5Mn (10 wt% Co-5wt% Mn/TiO_2 catalyst). Both scale bars are 30nm. (**c,d**) Nanoparticle size distributions for the (c) Co-0Mn and (d) Co-5Mn. For both distributions the mean diameter (**bold**), the standard deviation (σ) and the number of measurements (*n*) are given inset.

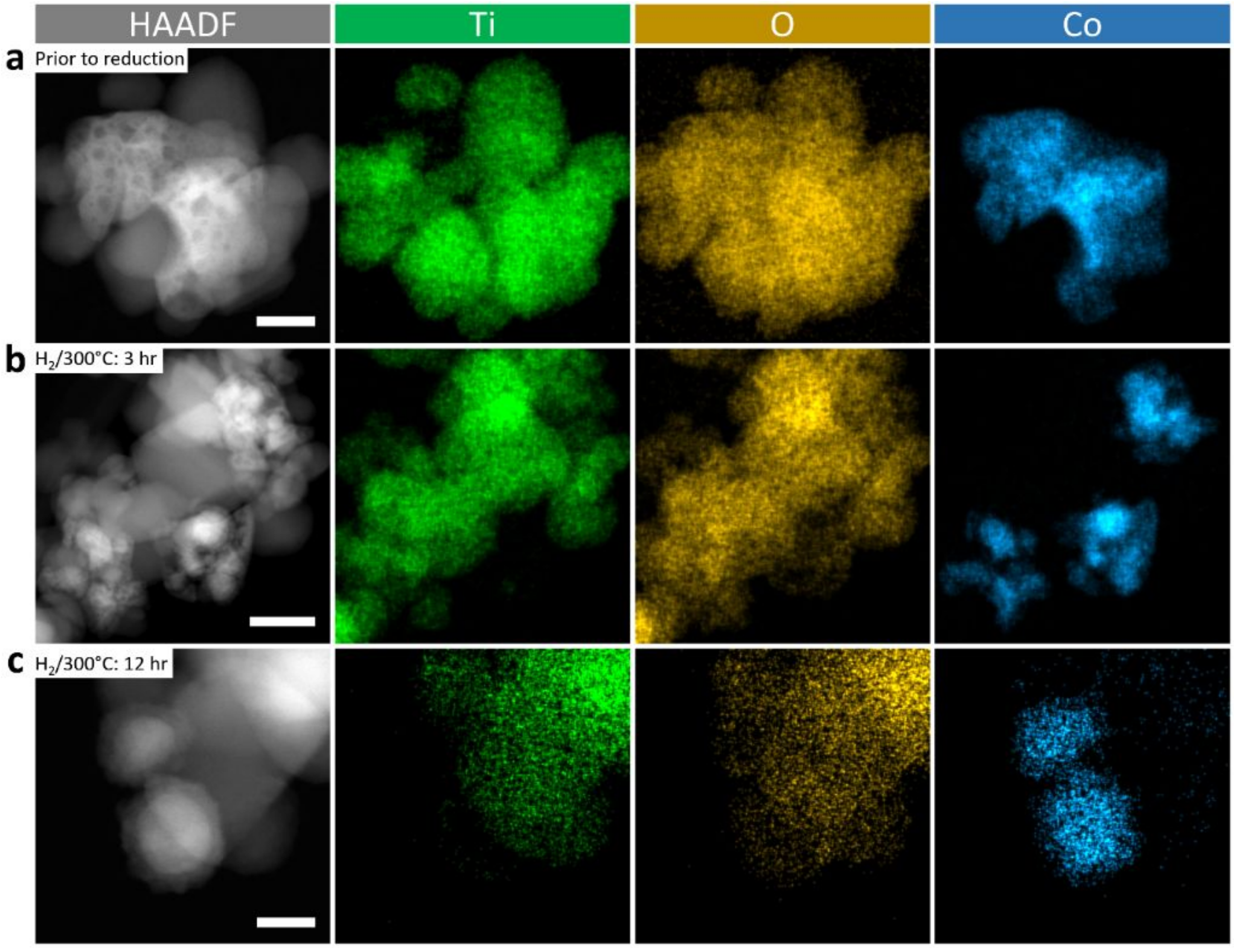

Figure S9: HAADF-STEM imaging and corresponding STEM-EDS elemental mapping of Co-0Mn (10wt% Co/TiO_2 catalyst) before and after ex situ activation. (**a**) The as calcined Co-0Mn sample (prior to activation) shows irregular shaped particles and porosity in Co-rich regions. Scale bar is 20 nm. (**b**) The Co-0Mn sample after it is subjected to ex situ activation for 3 hrs in 1 atm pure H_2. Scale bar is 30 nm. (**c**) The Co-0Mn sample after it is subjected to ex situ activation for 12 hrs in 1 atm pure H_2. Scale bar is 10 nm. The activation causes densification of the Co particles although at higher magnification the presence of an oxidised surface shell is visible, which likely formed due to accidental exposure to trace oxygen in the glovebox or to air during transfer to the microscope.

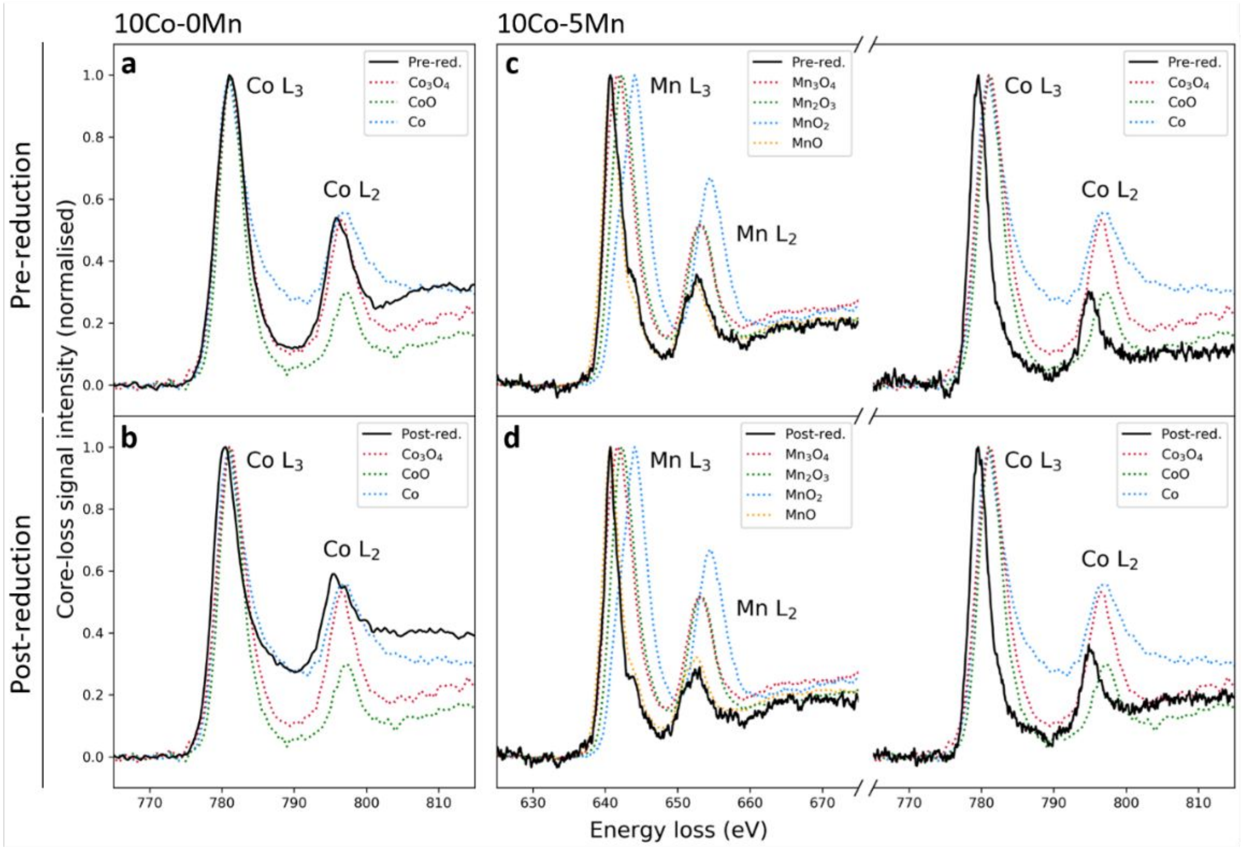

Figure S10: STEM-EELS analysis comparing the Co and Mn oxidation state before and after ex situ activation. (**a,b**) 10 wt% Co/TiO_2 and (**c,d**) 10 wt% Co-5 wt% Mn/TiO_2 catalysts pre- and post- *ex situ* reduction heat treatment, respectively, in H_2 (1 bar, 12hrs at 300°C). Summed STEM-EELS spectra from the catalysts (black) are compared to the measured spectra for the Co and Mn standards (dotted lines, legend provided on each spectra).

S2.4 In situ STEM of the catalyst morphology change during activation

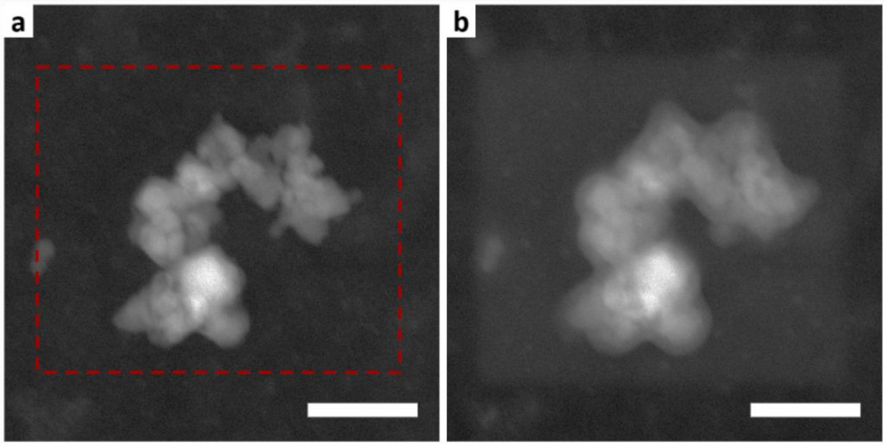

Figure S11: Example of contamination build up for Co-5Mn in the in situ gas cell (a) before and (b) after performing STEM-EDS mapping at 25°C. The STEM-EDS region of interest is highlighted by the red dashed rectangle in (a). In (b) a build-up of carbonaceous deposition at room temperature can be seen as the rectangle of increased intensity on the silicon nitride window and covering the surface of the nanoparticles. Scale bars are both 100 nm.

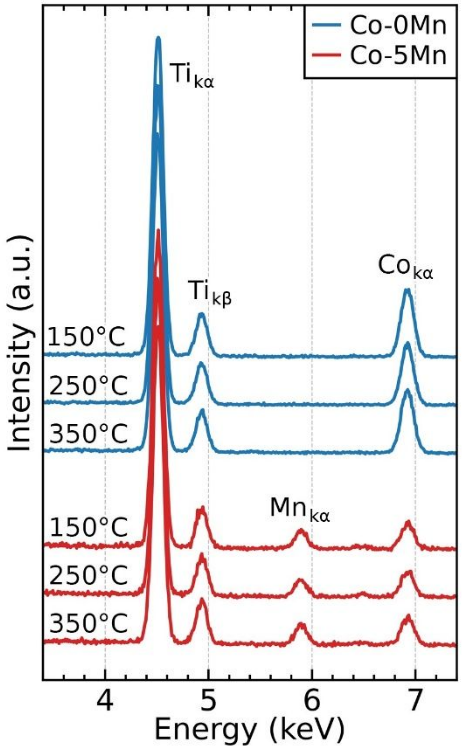

Figure S12: In situ STEM-EDS spectra of the catalysts. Corresponding spectra summed over the whole imaged regions in Figure 3a,b during the activation treatment. No change in the total Co (or Mn) content is observed during reduction.

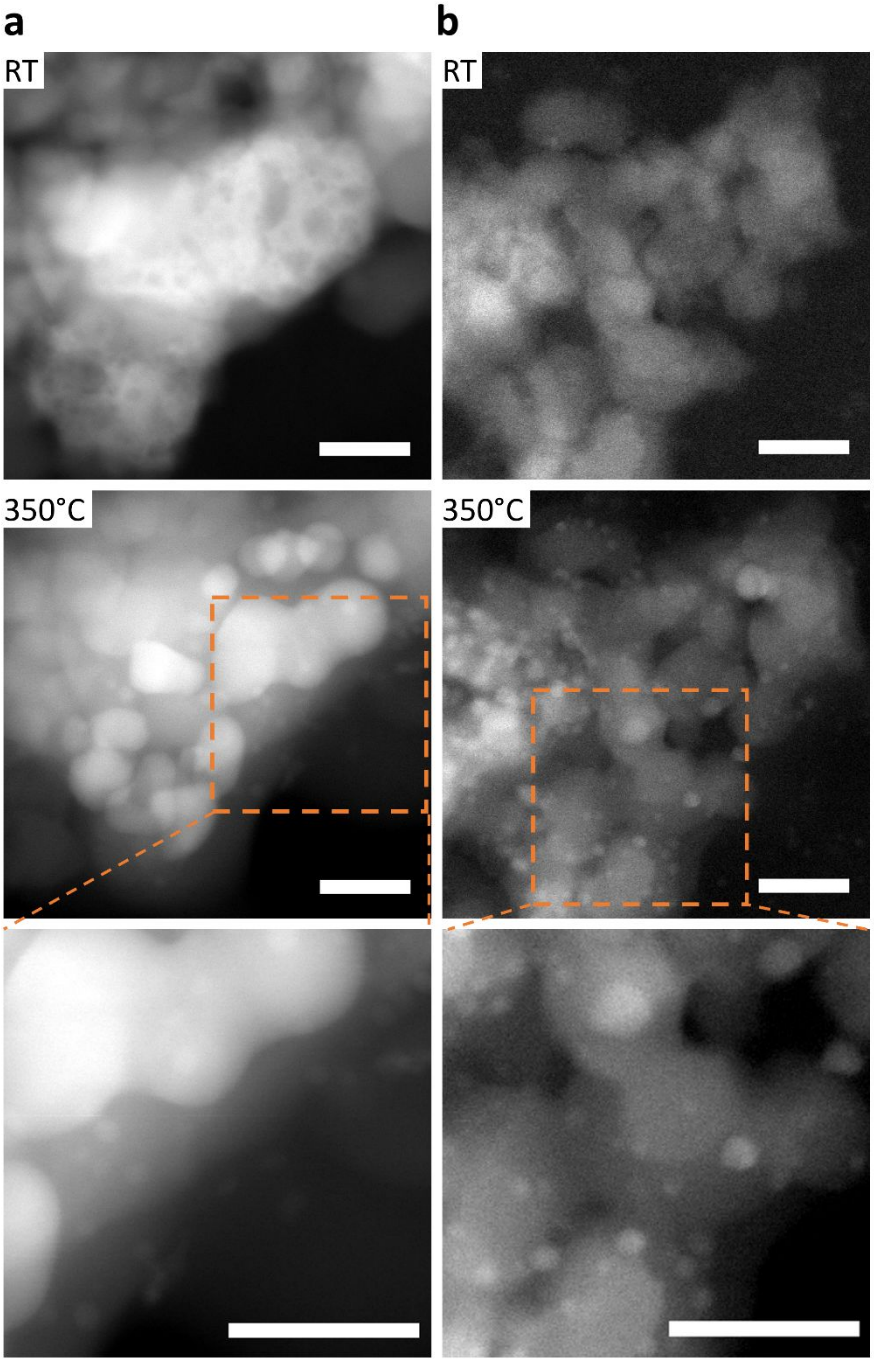

Figure S13: In situ HAADF-STEM imaging comparing the morphology of the catalysts imaged in H_2 at room temperature and at 350 C. (**a**) **Co-0Mn** (10 wt% Co/TiO_2 catalyst without Mn promotion), highlighting the readily distinguishable Co clustering and porous structure at room temperature (RT). Observations during increasing temperature to 350°C leads to particle sintering/coalescence (see Figure 4 for intermediate temperatures) and the formation of new much smaller paticles. (**b**) **Co-5Mn (**10 wt% Co-5 wt% Mn/TiO_2 catalyst) where the highly distributed Co and Mn is not visible from the HAADF STEM images at RT. The formation of denser nanoparticles likely to be the metal catalyst is observed at 350 C. Scale bars are all 30 nm.

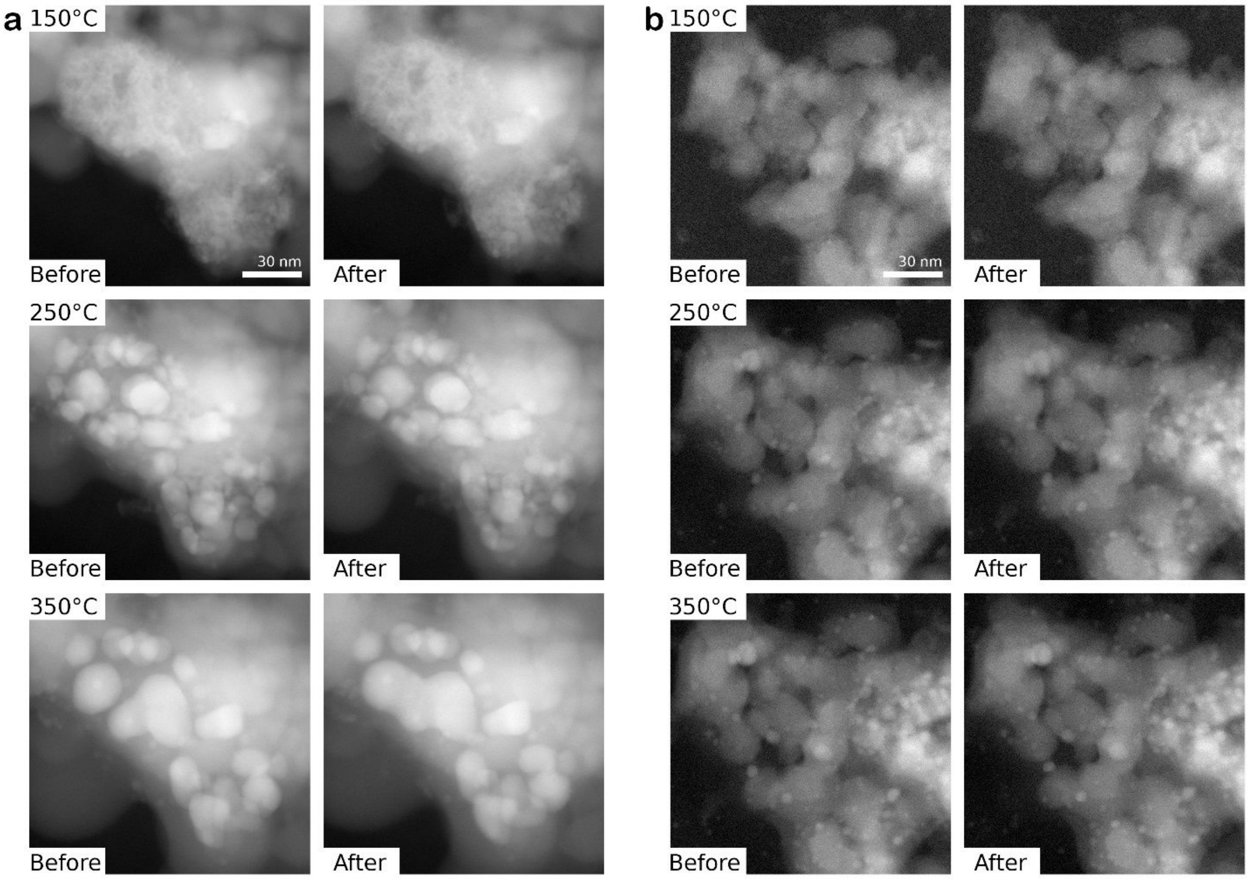

Figure S14: In situ HAADF STEM imaging of the regions in Figure 3 before and after EDS acqusition at each temperature step. The similarity between the images reveal the increased electron dose used during EDS did not contribute significantly to the sintering occuring as a result of the activation treatment.

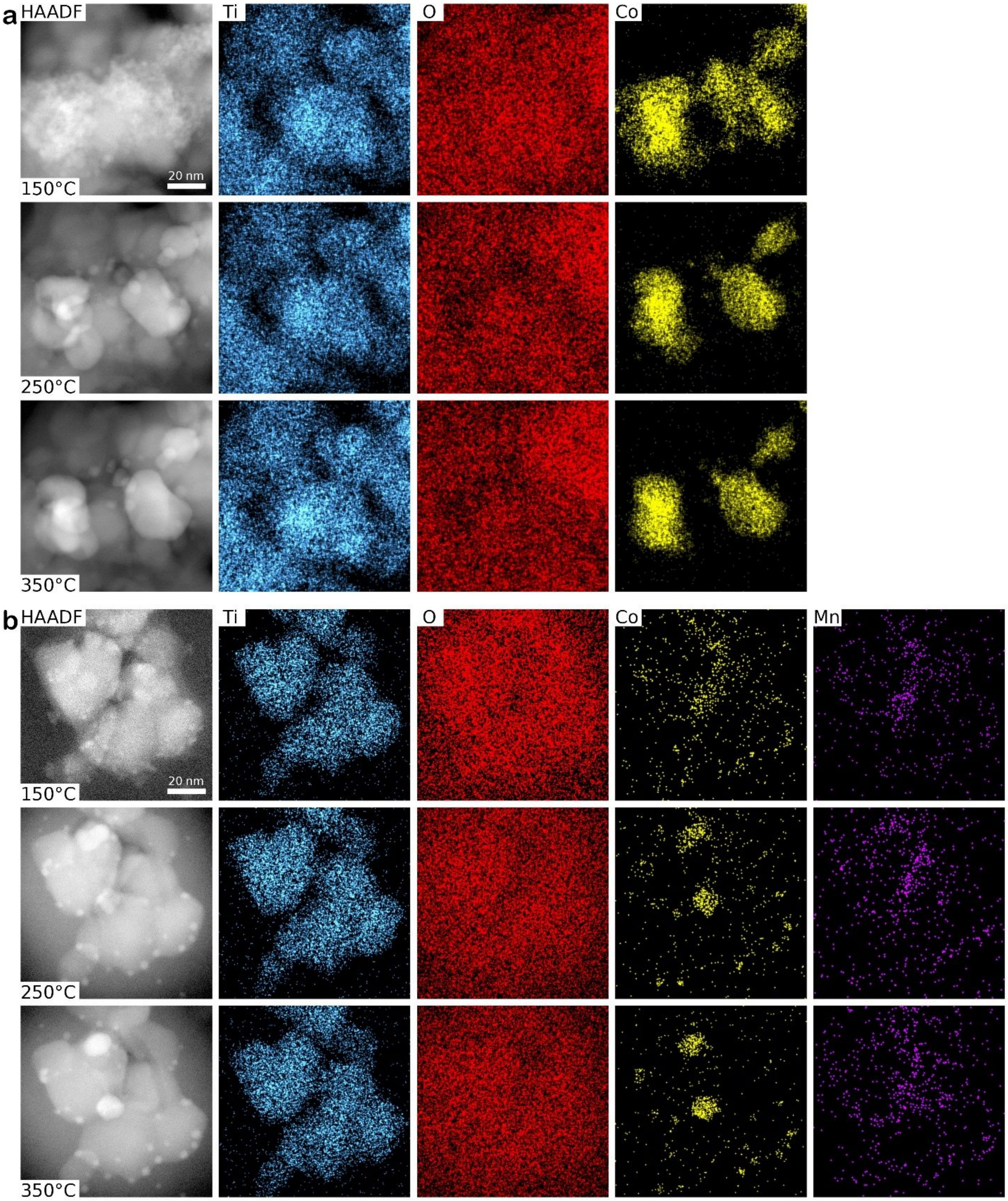

Figure S15: In situ structural and chemical characterisation of the TiO_2 supported Co/Mn catalyst dispersion during activation. (**a,b**) HAADF-STEM imaging and STEM-EDS elemental mapping of the (a) Co-0Mn and (b) Co-5Mn catalysts.

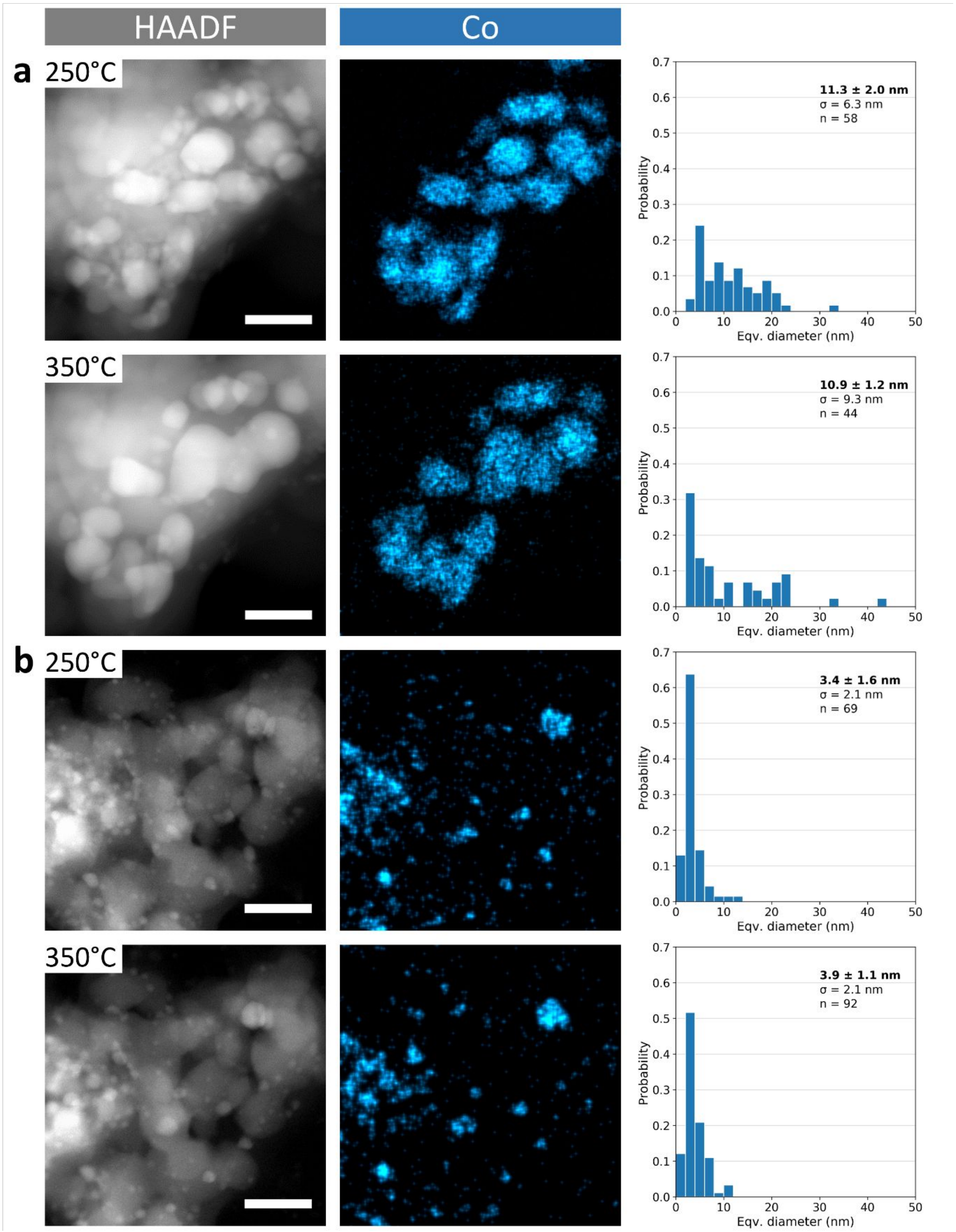

Figure S16: Analysis of Co particle size evolution during in situ STEM elemental analysis. (**a**) the 10 wt% Co/TiO_2 catalyst (without Mn promotion) and (**b**) 10 wt% Co-5wt.% Mn/TiO_2 catalyst. In each case the HAADF STEM image is presented (left) with the histogram of nanoparticle size distribution (right). Scale bars are all 30 nm. Sample areas are the same as shown in Figure 3a,b and Figure S7.

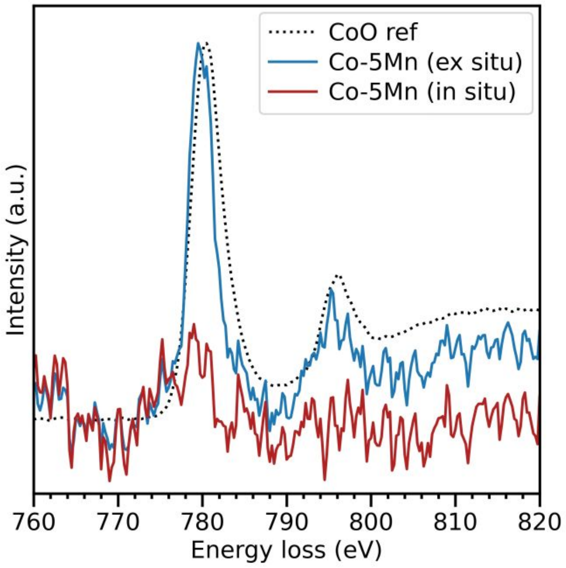

Figure S17: STEM-EELS of the Co edges in the Co-5Mn catalyst. Comparison of the summed spectra from ex situ and in situ observation using identical acquisition parameters illustrates the loss in signal-to-noise when collecting data in situ, which is due to the presence of the SiN_x membrane windows in the gas cell used to contain the environment within the vacuum of the TEM.

S2.5 X-ray Absorption Spectroscopy

In situ XANES/EXAFS have been used to investigate the oxidation state of the catalysts post-reduction in H_2 at 300°C for 8-hr. Figure S10a shows the XANES spectra of the Co edge for Co-0Mn and Co-5Mn after reduction in H_2 at 300°C for 8 hrs, normalised to a metallic Co foil standard. These samples are compared to CoO and to the 10wt% Co/TiO_2 catalysts with lower Mn loadings. For all catalyst samples a clear correlation can be seen to that of the Co metal reference, indicating the catalysts have been reduced to their active metallic state under these conditions. Quantitative estimation of the oxidation state using linear combination fitting (LCF) reveals the Co^0 (metal) content of the samples ranging from 94-98% (Table S1). A similar conclusion can be drawn from the extended x-ray absorption fine structure (EXAFS) spectra, where the catalyst profiles are closely aligned with the metallic Co foil reference (Figure S10b). The increase in Mn promotion also leads to a slight attenuation in the most intense peak at ~2.2 Å, associated with scattering from the Co-Co bond. This relative decrease is likely a result of the formation of smaller Co nanoparticles upon reduction, which serves to increase the surface area-to-bulk ratio and in turn the fraction of Co without full co-ordination. A slightly higher fraction of cobalt oxide was found to persist in the Co-5Mn compared to Co-0Mn after reduction.

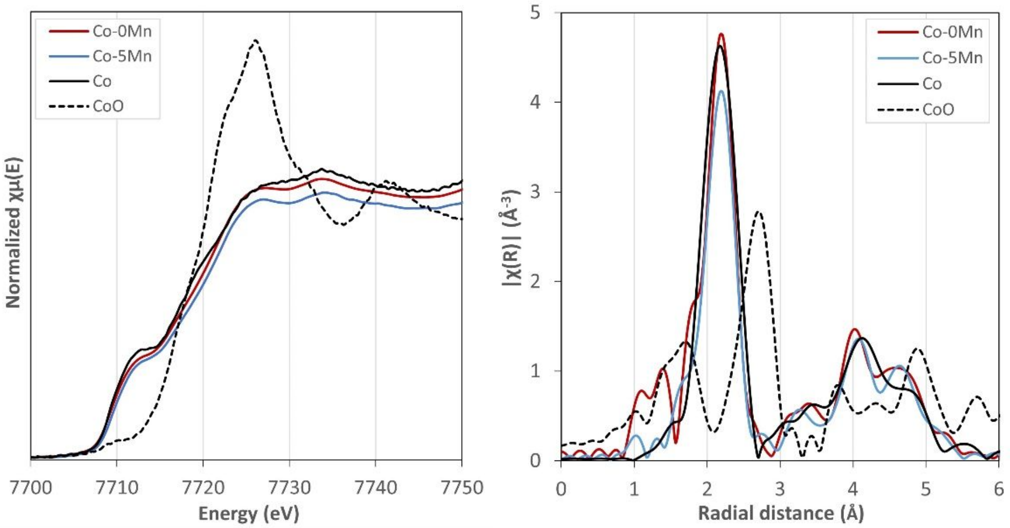

Figure S18: In situ XAS of the Co edge in 10wt% Co/TiO_2 Mn-promoted catalysts (1wt%, 2wt%, 3wt% and 5wt%) after reduction in H_2 at 300°C. The catalyst spectrum profiles are closely correlated to that of the reference metallic Co foil standard in both (**a**) XANES and (**b**) EXAFS.

Sample	Co^0 wt%	Co^{2+} wt%
Co-0Mn	98.3	1.7
Co-5Mn	96.8	3.2

Table S2: Quantitative oxidation state composition determined using linear combination fitting (LCF) of the XANES spectra calculates most of the cobalt to be in the metallic phase after reduction in H_2 at 300°C.

S2.6 Density Functional Theory

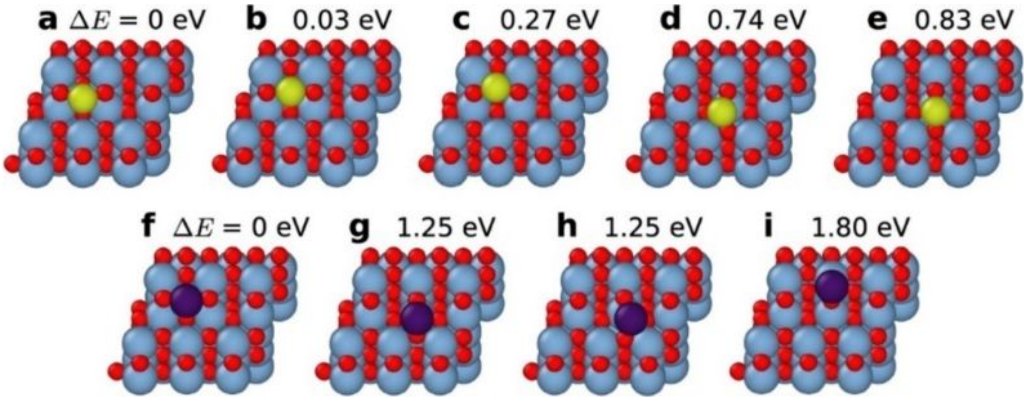

Figure S19: Most stable configurations for Co and Mn adatoms. The lowest energy configurations for (**a-e**) Co atom and (**f-i**) Mn atom adsorbed on the TiO_2 anatase (101) surface. Full structural details are provided in Table S2. Relative energies (ΔE) are given compared to the most stable configurations for Co (a) and Mn (f). Colours for Co, Mn, Ti, and O are yellow, purple, blue and red, respectively, analogous to the colours of the elemental maps in Figs. 1 and 2.

		E, eV	Total spin	Mulliken adatom spin	Mulliken adatom charge	Hirschfeld adatom charge	Distances to the nearest oxygen atoms, Å			
Co	a)	0.00	2	2.0	0.8	0.5	1.89	1.89	2.03	2.77
	b)	0.03	2	2.1	1.0	0.5	1.95	1.95	2.03	2.06
	c)	0.27	2	2.1	0.9	0.6	1.88	1.94	2.03	2.67
	d)	0.74	2	2.0	0.9	0.6	1.83	1.88	2.23	3.06
	e)	0.83	2	2.1	0.8	0.6	1.81	1.88	2.79	2.79
Mn	a)	0.00	5	4.8	1.1	0.9	1.91	1.91	2.13	2.41
	b)	1.25	4	4.5	1.0	0.8	1.87	1.95	2.22	3.12
	c)	1.25	6	5.9	0.7	0.6	2.01	2.27	3.42	3.42
	d)	1.80	5	5.6	0.5	0.5	1.98	3.28	3.31	4.12

Table S3. Characteristics of Co and Mn adatoms on anatase (101): relative energy, total spin, atomic spin and charge of metal adatom, distances to four nearest oxygen atoms.

Section 3: References

1. Harris, C. R., Millman, K. J., Van Der Walt, S. J., Gommers, R., Virtanen, P., Cournapeau, D., Wieser, E., Taylor, J., Berg, S., Smith, N. J., Kern, R., Picus, M., Hoyer, S., Van Kerkwijk, M. H., Brett, M., Haldane, A., Del Río, J. F., Wiebe, M., Peterson, P., Gérard-Marchant, P., Sheppard, K., Reddy, T., Weckesser, W., Abbasi, H., Gohlke, C. & Oliphant, T. E. Array programming with NumPy. *Nature* **585,** 357–362 (2020).
2. Virtanen, P., Gommers, R., Oliphant, T. E., Haberland, M., Reddy, T., Cournapeau, D., Burovski, E., Peterson, P., Weckesser, W., Bright, J., Van Der Walt, S. J., Brett, M., Wilson, J., Millman, K. J., Mayorov, N., Nelson, A. R. J., Jones, E., Kern, R., Larson, E., Carey, C. J., Polat, İ., Feng, Y., Moore, E. W., VanderPlas, J., Laxalde, D., Perktold, J., Cimrman, R., Henriksen, I., Quintero, E. A., Harris, C. R., Archibald, A. M., Ribeiro, A. H., Pedregosa, F., Van Mulbregt, P., SciPy 1.0 Contributors, Vijaykumar, A., Bardelli, A. P., Rothberg, A., Hilboll, A., Kloeckner, A., Scopatz, A., Lee, A., Rokem, A., Woods, C. N., Fulton, C., Masson, C., Häggström, C., Fitzgerald, C., Nicholson, D. A., Hagen, D. R., Pasechnik, D. V., Olivetti, E., Martin, E., Wieser, E., Silva, F., Lenders, F., Wilhelm, F., Young, G., Price, G. A., Ingold, G.-L., Allen, G. E., Lee, G. R., Audren, H., Probst, I., Dietrich, J. P., Silterra, J., Webber, J. T., Slavič, J., Nothman, J., Buchner, J., Kulick, J., Schönberger, J. L., De Miranda Cardoso, J. V., Reimer, J., Harrington, J., Rodríguez, J. L. C., Nunez-Iglesias, J., Kuczynski, J., Tritz, K., Thoma, M., Newville, M., Kümmerer, M., Bolingbroke, M., Tartre, M., Pak, M., Smith, N. J., Nowaczyk, N., Shebanov, N., Pavlyk, O., Brodtkorb, P. A., Lee, P., McGibbon, R. T., Feldbauer, R., Lewis, S., Tygier, S., Sievert, S., Vigna, S., Peterson, S., More, S., Pudlik, T., Oshima, T., Pingel, T. J., Robitaille, T. P., Spura, T., Jones, T. R., Cera, T., Leslie, T., Zito, T., Krauss, T., Upadhyay, U., Halchenko, Y. O. & Vázquez-Baeza, Y. SciPy 1.0: fundamental algorithms for scientific computing in Python. *Nat. Methods* **17,** 261–272 (2020).
3. Hunter, J. D. Matplotlib: A 2D Graphics Environment. *Comput. Sci. Eng.* **9,** 90–95 (2007).
4. Blum, V., Gehrke, R., Hanke, F., Havu, P., Havu, V., Ren, X., Reuter, K. & Scheffler, M. Ab initio molecular simulations with numeric atom-centered orbitals. *Comput. Phys. Commun.* **180,** 2175–2196 (2009).
5. Fricke, B. & Pershina, V. Atomic and Molecular Structure Calculations for Superheavy Elements. *J. Nucl. Radiochem. Sci.* **3,** 109–111 (2002).

6. Wellendorff, J., Lundgaard, K. T., Jacobsen, K. W. & Bligaard, T. mBEEF: An accurate semi-local Bayesian error estimation density functional. *J. Chem. Phys.* **140,** 144107 (2014).
7. Akimenko, S. S., Anisimova, G. D., Fadeeva, A. I., Fefelov, V. F., Gorbunov, V. A., Kayumova, T. R., Myshlyavtsev, A. V., Myshlyavtseva, M. D. & Stishenko, P. V. SuSMoST: Surface Science Modeling and Simulation Toolkit. *J. Comput. Chem.* **41,** 2084–2097 (2020).
8. Perdew, J. P., Burke, K. & Ernzerhof, M. Generalized Gradient Approximation Made Simple. *Phys. Rev. Lett.* **77,** 3865–3868 (1996).
9. Riva, R., Miessner, H., Vitali, R. & Del Piero, G. Metal–support interaction in Co/SiO2 and Co/TiO2. *Appl. Catal. Gen.* **196,** 111–123 (2000).
10. Feltes, T. E., Espinosa-Alonso, L., Smit, E. D., D'Souza, L., Meyer, R. J., Weckhuysen, B. M. & Regalbuto, J. R. Selective adsorption of manganese onto cobalt for optimized Mn/Co/TiO2 Fischer–Tropsch catalysts. *J. Catal.* **270,** 95–102 (2010).
